# Phytochemicals as NMDA Receptor Inhibitors and Their Potential for Treating Excitotoxicity-Related Neurotoxicity: A Systematic Review

**DOI:** 10.3390/cimb48060611

**Published:** 2026-06-11

**Authors:** Maryam N. ALNasser, Wayne G. Carter

**Affiliations:** 1 Department of Biological Sciences, College of Science, King Faisal University, P.O. Box No. 400, Al-Ahsa 31982, Saudi Arabia; malnasser@kfu.edu.sa; 2 Clinical Toxicology Research Group, School of Medicine, Royal Derby Hospital Centre, University of Nottingham, Derby DE22 3DT, UK

**Keywords:** excitotoxicity, neurodegenerative diseases, NMDA receptors, neuroprotection, phytochemicals

## Abstract

Excitotoxicity caused by excessive activation of glutamate receptors, particularly N-methyl-D-aspartate receptors (NMDARs), significantly contributes to neuronal damage in neurodegenerative diseases (NDDs), such as Alzheimer’s, Parkinson’s, and Huntington’s diseases. This systematic review aimed to evaluate the effects of plant extracts and phytochemicals on NMDAR-mediated excitotoxicity and to summarize their proposed neuroprotective mechanisms. The review protocol was registered in PROSPERO (CRD42024528160). A systematic search of Medline, Embase, Web of Science Core Collection, and PubMed identified 323 records, with an additional 7 records identified through manual searching that specifically considered in vitro and in vivo inhibitors of NMDAR excitotoxicity using plant extracts and isolated phytochemicals. Twenty-seven studies demonstrated that plant extracts and phytochemicals attenuate excitotoxicity through multiple mechanisms, including inhibition of NMDAR-induced currents, reduction of intracellular calcium influx, modulation of NMDAR expression, attenuation of oxidative stress, and mitochondrial dysfunction. However, the evidence base was largely dominated by in vitro and ex vivo studies, with limited in vivo validation, restricting translational relevance. Risk-of-bias assessment using an adapted version of the Office of Health Assessment and Translation (OHAT) Risk of Bias Tool indicated that 4 studies had a low overall risk of bias, 12 had low to moderate risk, and 11 were at moderate risk, with key limitations related to inadequate reporting of blinding, randomization, and allocation concealment. In contrast, exposure characterization, outcome assessment, and confounding control were generally strong across studies. Although the findings support the mechanistic neuroprotective potential of certain plant extracts and phytochemicals against NMDAR-mediated excitotoxicity, further well-designed in vivo and clinical studies are required to establish their therapeutic relevance for the treatment of neurodegenerative diseases.

## 1. Introduction

N-methyl-D-aspartate receptors (NMDARs) play a key role in regulating neuronal migration, synaptogenesis, maturation, and synaptic plasticity, all of which are essential for sustaining brain networks, cognition, and behavior [1]. Impaired NMDAR signaling can contribute to serious neurological disorders such as stroke, schizophrenia, and a number of neurodegenerative diseases (NDDs) [1]. NMDARs, which are heterotetrameric protein complexes composed of two GluN1 subunits combined with two additional subunits, either GluN2(A–D) or, less frequently, GluN3(A–B), facilitate excitatory postsynaptic signaling in the central nervous system (CNS) [1,2]. The glutamate-binding subunit of NMDARs is GluN2, while the glycine or D-serine-binding subunits are GluN1 and GluN3, such that NMDAR tetramers usually contain two GluN2 and two GluN1 [1,2].

Each NMDAR subunit is structured from two extracellular clamshell-shaped domains and includes an N-terminal domain (NTD) and an agonist binding domain (ABD), and these are the sites of action for allosteric modulators and agonists/co-agonists, respectively [1,2]. In addition, there is a transmembrane domain that forms the NMDAR channel pore and gate and includes four transmembranous helices: M1–M4. At the end of the subunit, there is an intracellular cytoplasmic carboxy-terminal domain that varies in length [1,2].

NMDAR activation is triggered by the simultaneous binding of glycine or D-serine with GluN1 subunits and glutamate (L-Glu) with GluN2 subunits, which shifts the conformation of the clamshells in the ABD to allow ion entry [1,3]. Postsynaptic membrane depolarization is essential for removing magnesium ions (Mg^2+^) or zinc ions (Zn^2+^) that trigger the closing of the NMDAR channel, resulting in the influx of sodium (Na^+^) and calcium (Ca^2+^) ions [1,3]. As a result, entry of these ions promotes the strength of synaptic connections by initiating intracellular signaling pathways [1]. However, the NMDAR channel formed by GluN1/GluN3 subunits has minimal influence on excitotoxicity due to its low Ca^2+^ influx, lack of modulation by the Mg^2+^ block, and reduced sensitivity to L-Glu [3,4].

Excitotoxicity occurs when L-Glu accumulates excessively in the extracellular space, triggering overactivity of NMDARs and leading to Ca^2+^ overload and neurodegeneration [3,5]. Neurodegeneration and neuronal death are likely to progress gradually and are linked to NDDs such as Alzheimer’s disease (AD), Parkinson’s disease (PD), multiple sclerosis (MS), amyotrophic lateral sclerosis (ALS), and Huntington’s disease (HD) [3,5]. Therefore, lowered L-Glu activity, either by regulating NMDAR binding or downstream activity or by clearing L-Glu from synapses, could provide a means to prevent excitotoxicity-induced neuronal death [3]. As a consequence of the impairment of astrocyte-neuron L-Glu clearance in several NDDs, moderating NMDAR activity remains a promising therapeutic approach for mitigating the degenerative process [3,6].

Many antagonists inhibit NMDARs, and these have been categorized based on their binding sites into non-competitive (channel blockers), competitive antagonists (which act at the Gly or L-Glu sites), and allosteric modulators (which act at the receptor NTD) [3,7,8]. Several NMDAR blockers have been used for treatment, including amantadine for PD, memantine for AD, and riluzole for ALS, although they can elicit undesired side effects such as cognitive impairment, hallucination, and psychosis [3,8]. However, unique NMDAR subunits are expressed in various brain regions, especially the GluN2 subunit, which raises the possibility of creating subunit-specific antagonists with more tolerable side effects [2].

Medicinal plants provide beneficial phytochemicals that could be exploited for their preferential safety, accessibility, affordability, established history of use, and low potential for adverse effects. Natural compounds found in plants, vegetables, fruits, and beverages could be used for treating neurological disorders [9,10,11]. In vitro and in vivo preclinical research, as well as clinical trials, have shown that plant extracts and their isolated phytochemicals have modulatory effects on CNS receptors [10]. Plant extracts and isolated phytochemicals could specifically act at NMDARs to provide novel treatments for neurological disorders and NDDs. Hence, this systematic research study was conducted to deliver an impartial analysis of whether plant extracts and isolated phytochemicals are capable of inhibiting NMDAR activation and the triggering of excitotoxic mechanisms.

## 2. Materials and Methods

### 2.1. Protocol Registration

This systematic review considered pre-clinical research from in vitro and in vivo studies and followed the Preferred Reporting Items for Systematic Reviews and Meta-Analyses (PRISMA) guidelines [12,13] (Supplementary Table S1). The study protocol was registered in the International Prospective Register of Systematic Reviews (PROSPERO) with registration number CRD42024528160.

### 2.2. Search Strategy

An electronic database search was carried out on 1 March 2026 using four databases, Medline (OvidSP), Embase (OvidSP), Web of Science Core Collection, and PubMed, in order to gather information about the potential inhibitory effects of plants and plant extracts on the excitotoxicity of NMDARs. Controlled search vocabularies (MeSH) were used and involved a combination of the following: (a) (In vitro OR human neuron) and (In vivo OR animals OR mammals) (b) Plants OR plant product OR plant extracts OR Herb OR herbal medicine. (c) Anti-excitotoxic* OR Anti-excitotoxicity OR L-glutamate toxicity inhibitor OR NMDA receptor antagonist OR N-methyl-D-aspartate receptor antagonist OR NMDA Receptor Blockade. Additional manual searches of related papers were conducted in relevant review papers and bibliographies. Search terms were applied over the period of 1 January 1999 until 1 March 2026.

### 2.3. Eligibility Criteria

All retrieved literature (*n* = 323) was exported into EndNote^®^ and Rayyan^®^ software (http://rayyan.qcri.org/, accessed on 7 May 2026) for further screening and duplicate removal. The studies were selected based on the eligibility criteria established by two independent researchers (M.N.A and W.G.C.). Titles and abstracts were used in the first screening phase to identify papers that met the predetermined inclusion criteria. After this, a full-text reading of each selected article was conducted before it was included in the final literature dataset.

#### 2.3.1. Inclusion Criteria

The results of all in vitro neuronal investigations that directly considered the possible inhibitory effect of plants and plant extracts on NMDAR excitotoxicity were included. Additionally, all in vivo (animal) studies that demonstrate an anti-excitotoxic effect of plant extracts were included. The two authors of this manuscript reviewed all papers that met the inclusion criteria and independently performed data extraction and discussed any anomalies.

#### 2.3.2. Exclusion Criteria

Studies in which plant products act on L-Glu receptors other than NMDARs were excluded, as well as studies including review articles, editorials, and abstracts submitted for conference presentations.

### 2.4. Data Extraction, Collection, and Synthesis

Data were extracted from eligible articles, and information was collected for the following variables: research title, author(s), year of publication, type of studies (in vitro or in vivo), type of cell or animal species, plant scientific name, type of extract, dose or concentration of plant extract or phytochemical and treatment groups, methods used, overall study outcomes, and conclusions. Study data from each of the articles was qualitatively synthesized into the main text.

A meta-analysis was not feasible due to the considerable heterogeneity among the extracted data. The studies included in this review demonstrated substantial methodological and clinical heterogeneity, which justified the use of a qualitative synthesis rather than meta-analysis. Variability was observed in experimental models, including in vitro neuronal cell lines, ex vivo brain tissue preparations, and a limited number of in vivo animal studies using different species and cellular systems. There was also marked diversity in the phytochemical interventions, ranging from crude plant extracts to isolated bioactive compounds, with differences in extraction methods, purity, and chemical characterization.

In addition, studies employed heterogeneous excitotoxic stimuli, including NMDA and L-Glu at varying concentrations, which were often supraphysiological and not standardized across experiments. Outcome measures also differed considerably, encompassing NMDAR-mediated currents, intracellular Ca^2+^ influx, oxidative stress markers, mitochondrial function, receptor expression levels, and neuronal viability assays. Differences in assay techniques, exposure times, and reporting formats further limited data comparability.

Overall, this methodological and experimental diversity across models, interventions, and outcome assessments prevented quantitative synthesis of the data and supported a narrative synthesis approach.

### 2.5. Risk of Bias Assessment

The methodological quality and internal validity of the included studies (*n* = 27) were assessed using an adapted version of the Office of Health Assessment and Translation (OHAT) Risk of Bias Tool [14]. Two reviewers independently conducted the assessments, with any disagreements resolved through consensus. The tool consists of 11 signaling questions across seven domains: selection bias (Q1–Q3), confounding (Q4), performance bias (Q5–Q6), attrition/exclusion bias (Q7), detection bias (Q8–Q9), selective reporting bias (Q10), and other sources of bias (Q11). The original OHAT questions were retained and supplemented with study-specific adaptations for NMDA receptor-modulating plant extracts and phytochemicals. The same framework was applied to both in vivo and in vitro studies (Supplementary Tables S2–S4).

Each item was rated as Definitely Low (++), Probably Low (+), Probably High (−), or Definitely High (−−) risk of bias, with a Not Applicable (NA) option used when a criterion was unsuitable for the study design.

Domain- and study-level ratings were generated separately for in vitro and in vivo studies, and an overall risk-of-bias judgment (low, low–moderate, or moderate) was assigned.

## 3. Results

**Figure 1:**
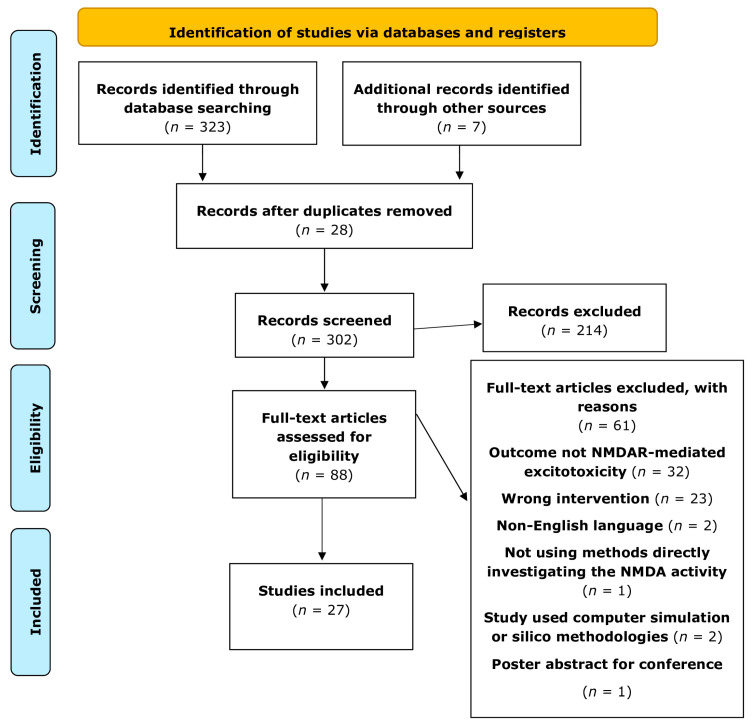
Preferred Reporting Items for Systematic Reviews and Meta-Analyses (PRISMA) flowchart showing the processes of data collection and selection [12,13].

A total of 323 articles were identified through the primary database search, with an additional 7 articles located through manual searching of relevant papers. Following the elimination of duplicates, 214 papers were excluded after screening their titles and abstracts, resulting in 88 articles for full-text evaluation. Of the 88 studies reviewed, 61 did not meet predefined eligibility criteria and were rejected based on the following reasons: outcome not NMDAR-mediated excitotoxicity (*n* = 32), wrong intervention (*n* = 23), written in a language that was not English (*n* = 2), did not use methods directly investigating NMDAR activity (*n* = 1), was a study that used computer simulation and/or a silico methodology (*n* = 2), and was a poster abstract for a conference (*n* = 1). Consequently, 27 articles met the criteria for inclusion. According to the PRISMA protocol, a flowchart was generated to show how studies were identified and selected (Figure 1). Most of the studies utilized in vitro methods (*n* = 23), while three used mixed methods (in vitro and in vivo) and one study was wholly undertaken in vivo.

Studies included in this review used a variety of models, including primary cortical neuronal cultures obtained from Sprague–Dawley rats (*n* = 7), mouse primary cortical neuronal cultures (*n* = 6), primary hippocampal neurons cultured from Sprague–Dawley or Wistar rats (*n* = 6), in vivo Sprague–Dawley or Wistar rats (*n* = 4), human neuroblastoma SH-SY5Y cells (*n* = 3), mouse primary hippocampal neurons (*n* = 1), mouse coronal brain slices (*n* = 1), mouse cortical wedge (*n* = 1), human embryonic kidney (HEK) 293 cells transfected with GluN1/GluN2A or GluN1/GluN2B subunits (*n* = 2), rat primary cerebellar granule cells (*n* = 1), *Xenopus* oocytes (*Xenopus laevis*) injected with total RNA prepared from Wistar rat cortices or cerebelli (*n* = 1), synaptic forebrains from Sprague–Dawley rats (*n* = 1), and differentiated human rhabdomyosarcoma (dTE671) cells (*n* = 1) (Table 1).

Studies were included only if they demonstrated a direct inhibitory effect of plant extracts or phytochemicals on NMDAR excitotoxicity. This was evidenced by the ability to block or inhibit NMDAR-induced currents, reduce NMDAR expression, block Ca^2+^ responses, diminish the levels of excitotoxicity-induced reactive oxygen species (ROS), mitigate excitotoxicity-induced mitochondrial dysfunction, or inhibit an excitotoxicity-related reduction of cell viability and/or apoptosis.

### 3.1. Plant Extracts and Isolated Phytochemicals Can Inhibit NMDAR-Induced Current

A total of 14 studies reported that plant extracts can inhibit NMDAR-induced currents in neuronal cells. Huperzine A, an alkaloid extracted from the Chinese herb *Huperzia serrata*, reversibly inhibited NMDA-induced currents with an IC_50_ of 126 μM, exhibiting non-competitive inhibition [16]. Rhynchophylline and isorhynchophylline, major tetracyclic oxindole alkaloids found in *Uncaria* species, inhibited NMDA-evoked current responses in a concentration-dependent manner at 1 to 100 μM in *Xenopus laevis* oocytes that expressed NMDAR from rat cortices or cerebelli [17], with IC_50_ values of 43.2 μM and 48.3 μM, respectively [17]. An isolate of *Ginkgo biloba* leaf extracts, including bilobalide at a concentration of 10 µM, slightly inhibited NMDA-mediated currents in NMDARs, whereas ginkgolide M strongly blocked NMDA-receptor-gated responses to NMDA exposure in rat hippocampal neurons [18]. Aqueous extracts of *Scutellaria baicalensis*, *Stephania tetrandra*, *Uncaria rhynchophylla*, and *Salvia miltiorrhiza* (1% *v*/*v*) inhibited NMDA-induced currents in mouse cortical neurons, and these extracts had varying concentrations of Mg^2+^: 12.5, 2.0, 0.8, and 8.7 mM, respectively [19]. Aqueous extracts (1 mg/mL) from dried roots of *Salvia miltiorrhiza* Bunge (Labiatae) blocked NMDA-evoked currents in mouse cortical neurons [20], although this may, at least in part, be due to the free Mg^2+^ ions present in the extracts. Mouse cerebral cortex slices treated with ethanolic extracts from *Searsia dentata* and *Searsia pyroides* (0.01–2.0 mg/mL) inhibited the NMDA antagonist [^3^H]-CGP39653, with IC_50_ values of about 0.48 and 0.73 mg dry extract/mL, respectively [25]. Ethanolic leaf extracts from the South African plants *Searsia dentata*, *Searsia pyroides*, and *Searsia glauca* (0.1–1 mg/mL) reduced the current induced by 500 μM NMDA and 30 μM glycine in rat cerebellar granule cells [28]. *Searsia dentata* and *Searsia pyroides* (0.1 mg/mL) blocked up to 70% of the current, with an ED_50_ of 0.022 mg/mL and 0.031 mg/mL, respectively, and this showed no significant recovery after prolonged washing [28]. The ethanolic extract of *Scutellaria baicalensis* Georgi (roots) competitively inhibited the binding of a selective antagonist for the glycine site of NMDAR ([^3^H]MDL 105,519) in a concentration-dependent manner, with an IC_50_ value of 35.1 μg/mL in the synaptic forebrain membrane of rats [31]. Furthermore, this plant ethanolic extract inhibited the binding of the NMDAR antagonist [^3^H]MK-801, with an IC_50_ value of 65.1 μg/mL in rat synaptic forebrain membranes [31]. Oxygen–glucose deprivation (OGD)-induced NMDAR-mediated currents were significantly inhibited by application of Hydroxysafflor yellow A (HSYA) (100 μM), a compound extracted from *Carthamus tinctorius* in mice brain slices [33]. Additionally, HSYA significantly reduced the amplitude of NMDAR-mediated excitatory postsynaptic currents (EPSCs) in a concentration-dependent manner (0.1 to 100 μM HSYA, with an IC_50_ value of 17.60 μM), in mouse coronal brain slices [33]. Further, 100 μM of HSYA significantly suppressed NMDAR-dependent OGD-induced ischemic long-term potentiation (iLTP) in brain slices [33]. In addition, an isolated bidentatide extract (100 nM) derived from *Achyranthes bidentata* Blume inhibited NMDA-evoked current in rat hippocampal neurons [39]. YY-23, a compound derived from the traditional antidepressant Chinese medicine *Rhizoma anemarrhenae*, inhibited NMDA-induced currents at a concentration of 3 μM in rat hippocampal neurons [34]. This isolated compound exhibited characteristics of a non-competitive antagonist with a half-maximal inhibitory concentration (IC_50_) of 2.8 μM [34]. There were four other plant extracts that significantly inhibited NMDA-induced depolarization through NMDARs, baicalein (4 μM) from *Scutellaria baicalensis*, ginkgolide A (300 μM) from *Ginkgo biloba*, and extracts from *Searsia dentata* and *Searsia pyroides* (0.01–2.0 mg/mL), tested in mouse cortical neurons [25,35,37]. Lastly, the aqueous extract of acai berry, at concentrations of 0.001, 1, and 1000 µg/mL, significantly suppressed the L-Glu- and glycine-activated currents in differentiated human rhabdomyosarcoma TE671 cells, producing reductions of 32%, 49%, and 50%, respectively [40].

### 3.2. Plant Extracts and Isolated Phytochemicals Can Down-Regulate the Expression of NMDARs

Six different plant extracts reduced the expression of the GluN2B subtype of NMDAR. The overexpression of the GluN2B subtype by NMDA stimuli was significantly decreased in rat cortex neurons by hyperoside, a flavonoid compound isolated from *Rhododendron ponticum* L. leaves [27]. The addition of flax lignan at concentrations of 1 and 10 μM reduced the overexpression of the GluN2B subtype of NMDARs induced by NMDA in mouse cortical neurons [30]. Similarly, 2S,3R-6-methoxycarbonylgallocatechin isolated from the ethanolic extract of Anhua dark tea significantly inhibited NMDA-induced expression of GluN2B in human SH-SY5Y neuroblastoma cells [36]. Two natural derivatized compounds isolated from *Arctium lappa* L. roots, 1,5-O-dicaffeoyl-3-O-[4-malic acid methyl ester]-quinic acid (MQA) and 4, 5-O-dicaffeoyl-1-O-[4-malic acid methyl ester]-quinic acid (DCMQA), both reduced GluN2B expression and increased GluN2A expression in human neuroblastoma SH-SY5Y cells exposed to NMDA [32,38]. Lastly, pretreatment of rats with epigoitrin, an alkaloid abundant in the traditional Chinese herb *Radix isatidis,* significantly reduced the increase in NMDAR subunit GluN2B expression in the cortex of rats treated with kainic acid [41].

### 3.3. Plant Extracts and Isolated Phytochemicals Can Reduce Intracellular Ca^2+^ Levels

Twelve studies reported that plant extracts were effective in countering the NMDAR-induced rise in intracellular Ca^2+^ levels. The NMDA-induced Ca^2+^ increase in cultured neurons was inhibited by hyperforin from St. John’s Wort (*Hypericum perforatum*) (10 µM) [22], a methanolic extract of *Smilacis chinae* rhizome (SCR) and its isolated compounds oxyresveratrol, resveratrol [23], an ethanolic extract from *Searsia pyroides* (0.02 and 0.1 mg/mL) [28], Flax Lignan [30], MQA (20, 40 µM/mL) and DCMQA (20 μM), chemicals derived from *Arctium lappa* L. roots [32,38], hydroxysafflor yellow A from *Carthamus tinctorius* (1–100 μM) [33], and bidentatide extract from *Achyranthes bidentata* Blume [39]. Furthermore, intracellular Ca^2+^ levels, which increase in response to L-Glu exposure, were reduced in cortical neurons by the active ingredient Notoginsenoside R1 (NTR1) from *Panax notoginseng* [26], as well as by (+)-ampelopsin A, γ-2-viniferin, and trans-ε-viniferin isolated from the leaves and stems of *Vitis amurensis* (Vitaceae) [29]. The methanolic extract of *Sanguisorbae radix* inhibited the H_2_O_2_-induced increase in intracellular Ca^2+^ in rat cortical neurons, with a concentration-dependent effect observed within the range of 10 to 50 µg/mL [24]. Likewise, the methanolic extract of SCR at concentrations of 10–50 µg/mL inhibited the amyloid-beta (25–35)-induced increase in intracellular Ca^2+^ in cortical neurons [21]. 4,5-O-Dicaffeoyl-1-O-[4-malic acid methyl ester]-quinic acid at a concentration of 20 μM significantly enhanced the NMDA-induced reduction in the expression of Ca^2+^/calmodulin-dependent protein kinase II-α in SH-SY5Y cells [38].

### 3.4. Plant Extracts and Isolated Phytochemicals Can Reduce Reactive Oxygen Species Production

There were nine studies that provided evidence that plant extracts can reduce potentially damaging reactive oxygen species (ROS). The methanolic extract of SCR at a concentration range of 10 to 50 µg/mL inhibited ROS production generated by NMDA or Aβ (25–35) in rat cortical neurons [21,23]. The production of ROS by NMDA stimuli in cultured cortical neurons was also inhibited by oxyresveratrol and resveratrol isolated from SCR [23]. The methanolic extract of *Sanguisorbae* radix at a concentration range of 10–50 µg/mL significantly reduced the ROS increase induced by hydrogen peroxide in cortical neurons [24]. 2*S*,3*R*-6-Methoxycarbonylgallocatechin isolated from Anhua dark tea significantly decreased ROS in NMDA-treated human SH-SY5Y neuroblastoma cells [36]. Likewise, reduced ROS levels were observed in L-Glu-treated mouse cortical neurons in the presence of NTR1, the main active ingredient of *Panax notoginseng*, when applied at a concentration range of 0.1–10 μM for 1 h [26]. The isolated compounds from the leaf and stem of *Vitis amurensis* (Vitaceae) (+)-ampelopsin A, γ-2-viniferin, and trans-ε-viniferin inhibited 500 μM Glu-induced ROS generation in a concentration-dependent manner in rat cortical neuron cultures [29]. NMDAR-mediated excessive neuronal nitric oxide synthase expression and ROS levels in SH-SY5Y cells were diminished by 4, 5-O-dicaffeoyl-1-O-[4-malic acid methyl ester]-quinic acid (DCMQA) from *Arctium lappa* L. roots [38]. The elevated expression of membrane protein-postsynaptic density protein 95 (PSD95) that activated nNOS production in conjunction with NMDAR signals was also inhibited via DCMQA in SH-SY5Y cells [38]. Aqueous and ethanolic extracts of acai berry (0.1–100 µg/mL) lowered ROS levels induced by L-Glu in dTE671 cells and in a concentration-dependent manner [40]. Epigoitrin at 50 mg/kg significantly reduced cortical ROS levels in kainic-acid-treated rats [41].

### 3.5. Plant Extracts and Isolated Phytochemicals Can Ameliorate Excitotoxicity-Induced Mitochondrial Dysfunction

Five of the plant extracts reduced the mitochondrial dysfunction induced by NMDAR excitotoxicity. The increase in mitochondrial membrane potential (MMP) generated by L-Glu exposure was prevented by (0.1–10 μM) notoginsenoside R1 (NTR1), the major active constituent in Panax notoginseng, in a concentration-dependent way in cortical neurons of ddY mouse embryos [26]. Hydroxysafflor yellow A from Carthamus tinctorius protected mitochondria from NMDA-induced damage by reducing mitochondrial swelling and fragmentation in hippocampal neurons [33]. DCMQA (20 μM) from Arctium lappa L roots significantly inhibited the NMDA-induced reduction in MMP levels in human SH-SY5Y cells [38]. Bidentatide (25–200 nM) from Achyranthes bidentata Blume significantly mitigated NMDA-induced MMP depletion in hippocampal neurons [39]. Co-incubation of acai berry aqueous and ethanolic extracts (0.001–10 µg/mL) with L-Glu mitigated the L-Glu-induced decrease in ATP and MMP levels in dTE671 cells [40].

### 3.6. Plant Extracts and Isolated Phytochemicals Can Inhibit Excitotoxicity-Related Cell Death and Apoptosis

Undesired excitotoxicity from NMDAR activation can reduce the viability of neurons. Fifteen studies considered assays that measured markers of cell viability, including cellular metabolic activity, cytolysis, DNA fragmentation, protein release, stress markers, and cell death via apoptosis. Aqueous extracts from *Uncaria rhynchophylla* prevented NMDA-induced cell death in mouse cortical neurons [19]. The methanolic extract of *Smilacis chinae* rhizome (SCR) (10–50 µg/mL) reduced Aβ (25–35)-induced apoptotic cell death and caspase-3 production in rat cortical neurons [21]. SCR at concentrations of 10, 30, and 50 µg/mL, along with its isolated compounds oxyresveratrol (at 1 and 10 µM) and resveratrol (at 10, 30, and 50 µM), significantly reduced NMDA-induced cell death in rat cortical neurons [23]. Additionally, SCR notably decreased infarction in the coronal sections of rats subjected to middle cerebral artery occlusion (MCAO) and mitigated MCAO-induced neuronal cell damage [23]. The methanolic extract of *Sanguisorbae* radix (SR) (10, 30, and 50 µg/mL) from *Sanguisorba officinalis* L. (Rosaceae) along with its isolated catechin (10, 50, and 100 µM) significantly reduced H_2_O_2_-induced apoptotic neuronal death in cortical neurons [24]. Additionally, SR diminished infarction and edema in rats subjected to MCAO [24]. Notoginsenoside R1 (NTR1) (0.1 to 10 μM) from *Panax notoginseng* prevented reductions in cell viability, LDH release, and the number of apoptotic cells induced by L-Glu (10 μM) and in a concentration-dependent manner [26]. NTR1 also restored decreased levels of Bcl-2 and increased levels of Bax in a concentration-dependent fashion in neurons exposed to L-Glu and protected cells expressing the NR1/NR2B subunits from cell death induced by 100 μM NMDA [26]. However, NTR1 did not provide protection to cells expressing the NR1/NR2A subunits, suggesting that NTR1 may specifically guard neurons against L-Glu cytotoxicity mediated by NMDAR composed of NR1/NR2B subunits rather than NR1/NR2A subunits [26]. The flavonoid hyperoside from *Rhododendron ponticum* L. leaves demonstrated concentration-dependent protection against NMDA-mediated cell death [27]. It also inhibited NMDA-induced apoptosis and exhibited anti-apoptotic properties by increasing Bcl-2 expression and reducing Bax expression in cultured cortical neurons [27]. (+)-Ampelopsin A, γ-2-viniferin, and trans-ε-viniferin from *Vitis amurensis* (Vitaceae) reduced Glu-induced neuronal death [29]. They also countered the Glu-induced reduction in Bcl-2 levels and the increase in Bax expression in rat cortical neuronal cultures [29]. NMDA-induced neuronal injury and apoptosis were reduced by flax lignan from flaxseed via regulation of the expression of the B-cell lymphoma/leukemia-2 (Bcl-2) and Bcl-2 associated X protein (Bax) and by increasing the ratio of Bax/Bcl-2 in mouse cortical neurons [30]. The ethanolic extract of *Scutellaria baicalensis* Georgi (roots) (1 to 100 μg/mL) reduced LDH levels induced by 350 μM Glu or NMDA [31]. It also alleviated neuronal swelling, dendrite breakage, and nuclear shape changes in a concentration-dependent manner in rat cortical neuronal cultures (IC_50_s of 60.01 μg/mL and 28.60 μg/mL for Glu and NMDA-induced excitotoxicity, respectively) [31].

1,5-O-Dicaffeoyl-3-O-[4-malic acid methyl ester]-quinic acid (MQA) from *Arctium lappa* L. roots (20 and 40 µM/mL) inhibited NMDA-induced cell death, LDH leakage, nuclear fragmentation, DNA condensation, and cell apoptosis in human SH-SY5Y cells [32]. It also decreased the Bax/Bcl-2 ratio, cytochrome c levels, and the protein and activity levels of caspase-3 and caspase-9 in response to NMDA [32]. MQA also significantly decreased the phosphorylation of ERK1/2, p38 MAPK, and JNK1/2 while restoring the phosphorylation of CREB, Akt, and GSK-3β that were reduced by NMDA treatment [32].

Pretreatment with hydroxysafflor yellow A (1 to 100 μM) from *Carthamus tinctorius* significantly reduced NMDA-induced apoptotic cell death and in a concentration-dependent manner [33]. Additionally, it decreased the NMDA-induced increase in LDH levels and the production of cleaved caspase-3 in hippocampal neurons, also in a concentration-dependent manner [33].

2S,3R-6-methoxycarbonylgallocatechin (5–20 μM) from the ethanolic extract of Anhua dark tea reduced NMDA-induced increases in LDH levels, cell apoptosis, Bax/Bcl2 ratio, and the expression of cleaved-Poly (ADP-ribose) polymerase (PARP) and cleaved caspase-3 [36]. It also diminished DNA condensation and nuclear fragmentation in NMDA-treated human SH-SY5Y cells [36]. In addition, it increased cell survival and growth via an increase in the expression of the activated form of alpha serine/threonine-protein kinase (phospho-Akt) and phosphorylated ERK1/2 [36].

4,5-O-Dicaffeoyl-1-O-[4-malic acid methyl ester]-quinic acid (DCMQA) (10, 20, and 40 μM) from *Arctium lappa* L. roots inhibited the NMDA-induced reduction in cell viability [38]. DCMQA prevented LDH release and morphological damage in SH-SY5Y cells exposed to NMDA, as evidenced by a reduction in the number of shrunken and rounded cells. Additionally, DCMQA suppressed NMDA-induced apoptosis by decreasing the Bax/Bcl-2 ratio, inhibiting the over-release of cytochrome c, and reducing the over-expression of caspase-3 and caspase-9 [38]. Bidentatide from *Achyranthes bidentata* Blume improved the Bcl-2/Bax ratio and cell viability and reduced nuclear shrinkage, the percentage of TUNEL-positive cells, and caspase-3 activity in rat hippocampal neurons exposed to 100 µM NMDA [39].

Acai berry extracts protected dTE671 cells from the L-Glu-induced loss of cell viability [40]. Pretreatment with epigoitrin (10 mg/kg) significantly reduced Fluoro-Jade B (FJB)-positive cells, indicating decreased kainic acid (KA)-induced cortical degeneration and with no FJB-positive cells observed in the prefrontal or entorhinal cortices at the higher dose of 50 mg/kg [41]. Epigoitrin also significantly increased neuronal nuclei expression compared with the KA group, suggesting protection against excitotoxic neuronal damage [41]. Additionally, pretreatment with epigoitrin significantly reduced neuronal injury and inhibited the increase of phosphorylated death-associated protein kinase 1 (p-DAPK1) and DAPK1 expression in the cortex of KA-treated rats [41].

### 3.7. Plant Extracts and Isolated Phytochemicals Can Inhibit NMDAR Activity Through Other Mechanisms

Some studies used alternative techniques to evaluate NMDAR inhibition using plant extracts and phytochemicals. Bilobalide (1–100 µM), a constituent of Ginkgo biloba, completely suppressed the NMDA-evoked release of choline, used as a marker of NMDAR stimulation, in hippocampal slices from Wistar rats, and in a concentration-dependent manner, with an IC_50_ of 2.3 µM [15]. Bilobalide also prevented the formation of lyso-phosphatidylcholine and glycerophosphocholine, indicating a reduction in the NMDA-induced phospholipase A_2_ activation triggered by NMDAR and Ca^2+^ influx [15]. In addition, intraperitoneal injection of bilobalide (20 mg/kg) for 1 h prior to NMDA infusion completely prevented both the NMDA-induced release of choline and its convulsive effects in Wistar rats [15]. Similarly, the NMDA-induced release of choline in cortical neurons was suppressed by hyperforin from St. John’s Wort (*Hypericum perforatum*) [22]. The methanolic extract of *Smilacis chinae* rhizome (10 to 50 µg/mL) blocked the Aβ (25–35)-induced increase in Glu release in rat cortical neurons [21]. The methanolic extract of *Sanguisorbae* radix (10 to 50 µg/mL) blocked the H_2_O_2_-induced increase in Glu release in cortical neurons and also reduced neurological deficit scores in a MCAO model of stroke [24]. Spontaneous epileptiform discharges of mouse cerebral cortical slices were inhibited with ethanolic extracts of *Searsia dentata* and *Searsia pyroides* (0.01 to 2 mg/mL), with an ED_50_ of 0.62 mg and 1.67 mg of dry extract/mL, respectively [25].

### 3.8. Risk of Bias

Across the 27 studies included in this review, the overall risk of bias ranged from low to moderate. Twelve studies were classified as having a low to moderate risk of bias, while four studies—Weichel et al., 1999 [15], Wang et al., 2016 [33], Zhang et al., 2016 [34], and ALNasser et al., 2025 [40]—achieved an overall low risk rating. These studies demonstrated strengths such as blinded outcome assessment, well-characterized exposures, and adequate experimental replication. The remaining 11 studies were rated as moderate risk, primarily due to insufficient reporting of measures to reduce performance bias rather than concerns related to detection bias or confounding (Table 2 and Table 3).

The strongest methodological domains were comparison group selection (Q3) and control of confounding (Q4), both of which were rated as Probably Low risk across all studies. In vitro investigations employed consistent cell models and passage numbers between experimental groups, while in vivo studies used comparable species and strains. Furthermore, key exposure characteristics—including botanical source, extraction solvent, and administered dose—were generally well described.

Exposure characterization (Q8) and outcome assessment (Q9) were also strong across the evidence base. Studies investigating purified and structurally defined compounds, such as huperzine A [16], rhynchophylline and isorhynchophylline [17], ginkgolides and bilobalide [15,18], hydroxysafflor yellow A [33], and timosaponin YY-23 [34], were rated Definitely Low risk. Similarly, studies employing objective instrumental techniques—including patch-clamp electrophysiology, recombinant HEK293-GluN recordings, and calcium imaging—demonstrated a low likelihood of detection bias. Standardized crude extracts and colorimetric viability assays generally received Probably Low risk ratings. Experimental conditions (Q5) were particularly robust in electrophysiological studies, where each recorded cell functioned as its own control, resulting in a Definitely Low risk assessment.

The most frequently identified limitation was performance bias associated with the absence of reported blinding procedures (Q6), which was rated Probably High across the studies. Selection-related domains represented the next most common source of concern. In vitro studies rarely reported random allocation of treatments to wells (Q1) and seldom addressed allocation concealment (Q2), leading to Probably High or, where not applicable, Not Applicable ratings. Similarly, none of the in vivo studies reported allocation concealment procedures.

Among the animal studies, randomization (Q1) was explicitly described in only one investigation, Chang et al., 2025 [41]. Reporting of attrition and exclusions (Q7) was complete in two in vivo studies; however, Ban et al., 2008 [23] and Nguyen et al., 2008 [24] did not fully account for animal mortality or exclusions during the experimental period.

Selective reporting (Q10) was consistently assessed as Probably Low risk. Studies generally reported complete concentration ranges, multiple outcome measures, and, where applicable, negative findings, such as the lack of efficacy of hyperforin in an in vivo model of focal cerebral ischemia [22]. Other threats to internal validity (Q11) were also typically rated Probably Low risk, reflecting the use of appropriate statistical analyses—commonly ANOVA with Tukey, Dunnett, or Bonferroni post hoc corrections—and the inclusion of at least three independent experiments. A Probably High risk rating was assigned only in cases where biological replication was limited or the definition of experimental replicates was unclear, as observed in Pedersen et al., 2008 [25] and Marchetti et al., 2011 [28].

## 4. Discussion

This systematic review highlights the potential of phytochemicals to provide a source of NMDAR inhibitors able to mitigate excitotoxicity mechanisms, often activated in NDDs. Collectively, experimental evidence indicates that phytochemicals exert protective effects through mechanisms including blocking NMDAR-induced currents, reducing NMDAR expression, and inhibiting calcium (Ca^2+^) influx and responses, mechanisms crucial for excitotoxic pathways. Additionally, these natural compounds counter cellular redox stress by diminishing the levels of ROS associated with excitotoxicity, showcasing their antioxidant properties and reducing the levels of deleterious ROS that can trigger apoptosis and cell death. They also counteract excitotoxicity-induced mitochondrial dysfunction, further enhancing their neuroprotective profile. Additionally, certain plant extracts suppress NMDAR activity through mechanisms such as preventing choline release and associated convulsive effects in rats, inhibiting the formation of lyso-phosphatidylcholine and glycerophosphocholine, and blocking increases in L-Glu release. They also reduced neurological deficit scores in rats subjected to the middle cerebral artery occlusion model of stroke. Collectively, these findings indicate that these natural compounds may be promising therapeutic options for further development and utilization in managing NDDs characterized by excitotoxic damage.

### 4.1. Plant Extracts and Isolated Phytochemicals Provide Neuroprotection by Blocking NMDAR Currents

There were 14 studies that showed that plant extracts can block NMDA-activated currents. Extracts from *Scutellaria baicalensis*, *Stephania tetrandra*, and *Uncaria rhynchophylla* demonstrated significant inhibition of NMDAR-induced currents, although varying Mg^2+^ concentrations may have contributed to these effects [19]. Similarly, the aqueous extracts of *Salvia miltiorrhiza* Bunge inhibited NMDA-evoked currents in mouse cortical neurons; however, this effect was likely influenced, at least in part, by the presence of free Mg^2+^ ions (~9 mM) within the extract [20]. Because Mg^2+^ is a well-established non-specific blocker of NMDAR channels, the presence of free ions in aqueous extracts could be considered a confounding factor and methodological limitation that contaminates a validated pharmacological mechanism of the extracts themselves [42,43]. Therefore, caution is required when interpreting the observed neuroprotective effects of extracts containing relatively high ionic concentrations.

The isolated bidentatide extract from *Achyranthes bidentata* inhibited NMDA-evoked currents in rat hippocampal neurons, suggesting that specific phytochemicals can act as potent modulators of NMDAR activity [39]. Similarly, ethanolic extracts from *Searsia dentata*, *Searsia pyroides*, and *Searsia glauca*, along with the major alkaloids rhynchophylline and isorhynchophylline from *Uncaria* species, inhibited NMDA responses in a concentration-dependent manner [17,28]. In addition, hydroxysafflor yellow A significantly inhibited NMDAR currents induced by oxygen–glucose deprivation [33]. L-Glu-induced NMDAR currents were significantly inhibited by acai berry aqueous extracts, and several phytochemicals identified in acai berry extracts (histidine, arginine, 2,5-dihydroxybenzoic acid, threonine, and protocatechuic acid) were predicted to display relatively strong binding affinity to NMDARs to act as antagonists [40]. Hence, plants can harbor a range of compounds that could be neuroprotective and combat excitotoxicity through specific targeting of NMDARs [44].

Extracts from *Ginkgo biloba*, including bilobalide and ginkgolide M, exhibited varying degrees of inhibition on NMDA-mediated currents [18]. Additionally, YY-23 from *Rhizoma anemarrhenae* and Huperzine A from *Huperzia serrata* exhibited non-competitive antagonist properties, highlighting the diverse mechanisms by which plant extracts can modulate NMDAR activity [34]. The ethanolic extract of *Scutellaria baicalensis* showed competitive inhibition at the glycine site of NMDAR [31], and, similarly, competitive inhibition was observed with extracts from *Searsia dentata* and *Searsia pyroides*, which effectively inhibited NMDA antagonists in cortical neurons [25].

These varying levels of NMDAR modulation may be attributed to the distinct chemical structures, binding sites, and mechanisms of action of the isolated compounds. Furthermore, plant extracts may interact with multiple sites on NMDARs, which collectively modulate receptor activity. Notably, several well-characterized antagonists of NMDARs, such as ketamine, memantine, and dizocilpine maleate (MK-801), exert their effects through distinct binding sites and mechanisms [3]. For example, ketamine and MK-801 are non-competitive high-affinity antagonists that bind within the ion channel pore, blocking calcium influx when the receptor is activated [3]. In contrast, memantine exhibits a lower-affinity, voltage-dependent block, allowing it to preferentially inhibit excessive receptor activation while sparing normal synaptic transmission [3]. Other antagonists, such as D-AP5 (2-amino-5-phosphonopentanoate), function as competitive inhibitors at the L-Glu-binding site, whereas compounds such as ifenprodil selectively act at allosteric sites by binding to the GluN2B subunit interface within the N-terminal domain [3]. Thus, based on these established mechanisms, it is reasonable to suggest that bioactive compounds found in plant extracts may also bind to different regions of NMDARs, producing similar effects such as partial inhibition, subtype selectivity, or state-dependent blockade. Additional studies are required to confirm exact binding sites and better understand how these compounds affect NMDAR structure and function.

### 4.2. Plant Extracts and Isolated Phytochemicals Provide Neuroprotection by Altering NMDAR Expression

Plant extracts and their isolated compounds can influence NMDAR functionality by altering the expression of the GluN2B subtype of NMDARs, particularly in relation to NMDA-induced overexpression. Six different plant extracts reduced GluN2B expression [27,30,32,36,38,41]. Furthermore, compounds isolated from *Arctium lappa* L. roots also increased GluN2A expression [32,38]. NMDAR over-activation, particularly the GluN2B subtype, can lead to excitotoxicity and neuronal damage [45,46]. Hence, reduced GluN2B expression and increased GluN2A expression could have an impact on various NDDs and may provide a protective mechanism that counters excitatory signaling. In support of this suggestion, GluN2A activation links to cellular signaling pathways that protect neurons from damage, while the inhibition of NR2B-containing receptors also contributes to neuroprotection [47]. Therefore, these findings suggest that phytochemicals that can target the balance of GluN2B and GluN2A expression could be a promising strategy for neuroprotective interventions.

### 4.3. Plant Extracts and Phytochemicals Provide Neuroprotection by Altering Calcium Influx

Plant extracts and phytochemicals were able to reduce intracellular Ca^2+^ levels that were elevated in response to NMDAR over-stimulation from various neurotoxic stimuli. The extracts and isolated compounds from *Salvia miltiorrhiza* Bunge, *Achyranthes bidentata* Blume, *Searsia pyroides*, *Carthamus tinctorius*, *Hypericum perforatum*, *Smilacis chinae* rhizome (SCR), and *Arctium lappa* L. all lowered Ca^2+^ levels after NMDA exposure [20,22,23,28,32,33,38,39]. Additionally, *Panax notoginseng* and *Vitis amurensis* lowered Ca^2+^ levels in neurons following Glu exposure [26,29]. *Sanguisorbae radix* and SCR extracts reduced Ca^2+^ levels induced by H_2_O_2_ and Aβ (25–35), respectively [21,24], and 4,5-O-Dicaffeoyl-1-O-[4-malic acid methyl ester]-quinic acid enhanced the CaMK II-α expression, critical for Ca^2+^ homeostasis [38]. A reduction in intracellular Ca^2+^ levels is crucial because uncontrolled elevation of Ca^2+^ contributes to excitotoxicity, ultimately resulting in neuronal damage [48]. These findings suggest that certain phytocompounds can be neuroprotective by lowering excitotoxic Ca^2+^ levels directly but also stabilize calcium regulatory mechanisms.

### 4.4. Plant Extracts and Phytochemicals Provide Neuroprotection by Reducing Cellular Redox Stress

The neuroprotective effects of certain plant compounds were in part a reflection of their ability to reduce excessive ROS production, generated by the overactivation of NMDARs in response to neurotoxic stimuli. Overactivation of NMDARs increases cytosolic Ca^2+^ levels, which subsequently activate the arachidonic acid second messenger system and nitrogen oxide (NOX) (NADPH Oxidase), leading to the excessive production of ROS [49,50]. Plant extracts and isolated compounds, such as 2S,3R-6-methoxycarbonylgallocatechin, the methanolic extract of Sanguisorbae chinae rhizome (SCR), along with its isolated compounds oxyresveratrol and resveratrol, and DCMQA, reduced NMDA-induced ROS production [23,36,38]. Additionally, DCMQA reduced the NMDA-mediated increased expression of neuronal nitric oxide synthase (nNOS) and the membrane protein postsynaptic density protein 95 (PSD95), which activates nNOS through NMDAR signaling, in SH-SY5Y cells [38]. A reduction in ROS production in L-Glu–treated neurons was also observed following treatment with acai berry extracts and the isolated compounds NTR1, (+)-ampelopsin A, γ-2-viniferin, trans-ε-viniferin, and epigoitrin [26,29,40,41]. Excess ROS induced by H_2_O_2_ and Aβ (25–35) in cortical neurons was inhibited by *Sanguisorbae* and SCR, respectively [21,24]. These findings suggest that plant-derived compounds, which are often potent antioxidants, can mitigate damaging oxidative stress by reducing ROS levels, contributing to their potential neuroprotection effects.

### 4.5. Plant Extracts and Phytochemicals Provide Neuroprotection by Maintaining Mitochondrial Integrity

Certain plant extracts and phytochemicals were able to mitigate the mitochondrial dysfunction induced by NMDAR excitotoxicity. The overactivation of NMDARs leads to increased intracellular Ca^2+^ concentrations, disruption of the MMP and typically mitochondrial depolarization, and calcium overload in both the cytosol and mitochondria [51]. Plant-derived compounds, such as bidentatide crude extract and DCMQA, mitigated NMDA-induced depletion of the MMP in neurons [38,39]. Additionally, NTR1 countered an increase in the MMP associated with Glu exposure [26]. Hydroxysafflor yellow A protected mitochondria from NMDA-induced damage by reducing mitochondrial swelling and fragmentation in hippocampal neurons [33]. Acai berry extracts improved the ATP and MMP decline induced from L-Glu exposure [40]. These findings highlight the potential of plant-derived compounds to safeguard mitochondrial function in the context of excitotoxicity.

### 4.6. Plant Extracts and Phytochemicals Provide Neuroprotection by Maintaining Neuronal Integrity

Plant extracts were able to alleviate the detrimental effects of NMDAR activation on neuronal proliferation and viability, as measured by various assays evaluating metabolic activity, DNA fragmentation, and markers of apoptosis. Flax lignan from flaxseed reduced neuronal injury by regulating Bcl-2 and Bax expression and improving the Bax/Bcl-2 ratio [30]. Similarly, bidentatide and SCR enhanced cell viability and reduced apoptosis in neurons by lowering caspase-3 activity in NMDA-treated and Aβ-treated cells, respectively [21,39]. Other compounds, such as hyperoside from *Rhododendron ponticum*, NTR1 from *Panax notoginseng*, and (+)-ampelopsin A, γ-2-viniferin, and trans-ε-viniferin from *Vitis amurensis*, exhibited protective effects by increasing Bcl-2 expression and reducing Bax expression [26,27,29]. DCMQA from *Arctium lappa* L. inhibited NMDA-induced cell death and apoptosis, while 2S,3R-6-methoxycarbonylgallocatechin from Anhua dark tea improved cell survival by enhancing p-Akt and p-ERK1/2 levels [36,38]. Hydroxysafflor yellow A from *Carthamus tinctorius, Scutellaria baicalensis,* and MQA from *Arctium lappa* L. roots also reduced NMDA-induced apoptotic cell death [31,32,33]. Other extracts, such as those from *Smilacis chinae* and *Sanguisorba officinalis*, not only mitigated NMDA or H_2_O_2_-induced cell death but also diminished infarction in MCAO animal models [23,24]. Other plant-derived compounds also displayed neuroprotective effects: acai berry extracts and epigoitrin protected neurons from cell damage induced by L-Glu and KA, respectively [40,41]. Collectively, these findings suggest that various plant extracts and their active components can combat excitotoxicity-related neuronal damage and death by modulating the induction of apoptosis and associated signaling pathways.

Some studies employed assays to track the release of specific markers, such as choline, which serve as indicators of NMDAR activation. Bilobalide from *Ginkgo biloba* suppressed NMDA-evoked choline release in neurons and also prevented the formation of lyso-phosphatidylcholine and glycerophosphocholine [15]. Additionally, intraperitoneal injection of bilobalide before NMDA infusion blocked both choline release and convulsive effects in vivo [15]. Similarly, hyperforin from St. John’s Wort inhibited NMDA-induced choline release in cortical neurons [22]. Ethanolic extracts of *Searsia dentata* and *Searsia pyroides* inhibited SEDs in mouse cortical slices, highlighting their potential to modulate excitatory signaling [25]. Methanolic extracts of *Sanguisorbae radix* and *Smilacis chinae* rhizome blocked the Glu release induced by H_2_O_2_ and Aβ (25–35), respectively [21,24]. These findings evidence the neuroprotective effects of various plant extracts against NMDAR-related downstream excitotoxicity. Hence, collectively, targeting of NMDARs as well as downstream NMDAR signaling pathways represent a potentially fruitful means to limit damaging excitotoxicity, such as that observed in stroke [52].

### 4.7. Evaluation of the Risk of Bias

The risk-of-bias assessment indicated that the current evidence base is of generally acceptable quality for early-stage mechanistic pharmacology research, supporting confidence in the reported effects of NMDA receptor-modulating phytochemicals. Strengths were evident in the comparability of experimental groups, control of confounding variables, and the use of well-characterized exposures and objective outcome measures, all of which reduce the likelihood that observed effects were attributable to methodological artefacts. The widespread use of objective techniques, such as electrophysiology and calcium imaging, further strengthens confidence in the validity of the findings.

The principal methodological limitation was the inadequate reporting of measures to minimize performance and selection bias, particularly blinding, randomization, and allocation concealment. Although these omissions reduce confidence in the internal validity and reproducibility of individual studies, they appear to reflect common reporting practices within preclinical neuroprotection and in vitro pharmacology research rather than evidence of substantial methodological weaknesses. The consistently low risk of selective reporting and the use of appropriate statistical analyses further support the credibility of the evidence.

Overall, the findings suggest that the available evidence is sufficiently robust to support mechanistic conclusions regarding the neuroprotective potential of NMDA receptor-modulating phytochemicals. Nevertheless, future studies should adopt and clearly report bias reduction strategies, including preregistered randomization procedures, blinded outcome assessment, and comprehensive reporting of replication, attrition, and exclusions, to enhance transparency, reproducibility, and translational relevance.

### 4.8. Study Translational Limitations

A key limitation of several of the reported studies is the use of plant extracts rather than isolated compounds without identification of the specific active constituents responsible for NMDAR modulation. While this approach is useful as a first step in characterizing bioactivity, further experimentation is required to isolate and validate the individual compounds responsible, particularly for potential therapeutic development. In addition, the presence of potential contaminants in extract preparations, such as divalent metal cations (e.g., Mg^2+^), may have influenced the observed effects. Another methodological limitation is that experimental evidence derived from plant extracts and isolated phytochemicals did not consistently include concentration–response analyses. Without such data, it is difficult to establish pharmacological potency and reproducibility.

A further important limitation relates to the reporting of relatively weak in vitro potency of several natural compounds when compared to clinically established NMDAR antagonists. For example, Huperzine A exhibited an IC_50_ of approximately 126 µM for NMDAR inhibition [16], and ginkgolide A demonstrated neuroprotective effects only at concentrations near 300 µM [37]. These concentrations are substantially higher than those reported for therapeutic NMDAR antagonists such as memantine and ketamine, which typically act in the low micromolar range [53,54]. Importantly, the effective concentrations reported for several phytochemicals may exceed achievable CNS levels under standard pharmacokinetic conditions, raising concerns regarding translational feasibility and clinical relevance.

The evidence base is further limited by the predominance of in vitro and ex vivo models, which do not fully replicate physiological or pathological conditions. Variability in experimental design, including the use of non-physiological agonist concentrations (e.g., L-Glu), also reduces comparability between studies. In addition, the relatively small number of in vivo studies limits the strength of conclusions that can be drawn regarding therapeutic potential.

Translational challenges also remain, including uncertain blood–brain barrier penetration, bioavailability, metabolic degradation, and species differences in NMDAR expression and signaling pathways, all of which may limit extrapolation to humans.

Finally, the risk-of-bias analysis indicates that reporting of key bias reduction measures was generally limited across the included studies, particularly for blinding, randomization, and allocation concealment. As a result, it is often unclear to what extent such methods were implemented, which introduces uncertainty regarding the internal validity of the primary data. Because the conclusions of this systematic review are dependent on the quality of the underlying studies, these limitations should be considered when interpreting the findings and their translational relevance.

Overall, although the current evidence provides meaningful mechanistic insights and suggests potential neuroprotective effects, further in vivo, pharmacokinetic, mechanistic, and clinical investigations are required to strengthen and validate their therapeutic relevance in human NDDs.

### 4.9. Summary

**Figure 2:**
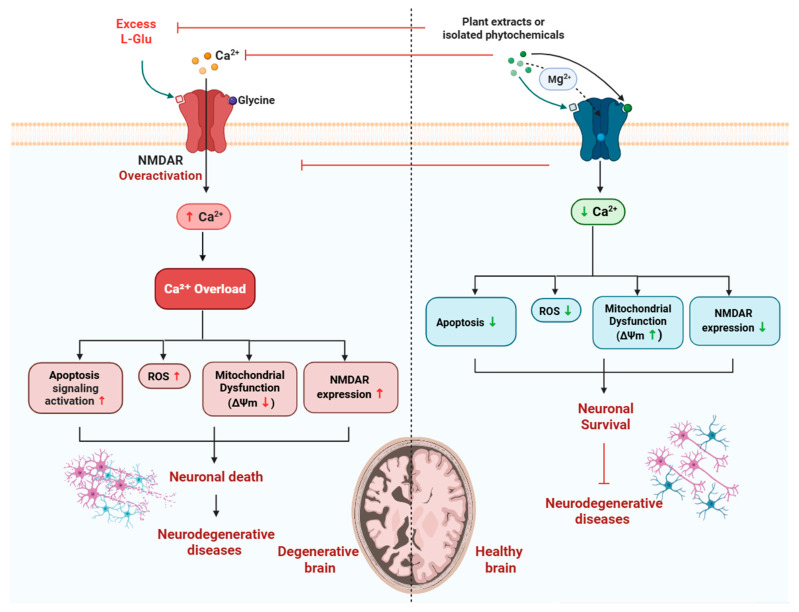
Summary of the neuroprotective effects of the plant extracts and phytochemicals against NMDAR-mediated excitotoxicity.

This systematic review highlights the potential of natural plant extracts and phytochemicals to act as inhibitors of NMDAR activation to combat excitotoxicity, a pathological mechanism common to a number of NDDs. Phytochemicals exhibited a range of protective mechanisms, including inhibition of NMDAR-induced currents, altered NMDAR expression, reduced calcium responses and ROS generation, restoration of mitochondrial membrane integrity (Figure 2), as well as blocking downstream effects such as choline release and PLA_2_ activation. Notwithstanding their therapeutic promise, there is a need for further exploration and dissection of specific active compounds and more detailed investigation into their mechanisms of action and optimal concentrations and dosages before in vivo translation can be considered. Furthermore, subsequent evaluation of the efficacy of these natural inhibitors in clinical settings is required to explore their potential, perhaps in combination with existing therapies to enhance neuroprotection against and treatment of excitotoxicity.

Excess L-glutamate (L-Glu) can trigger NMDAR overactivation, resulting in increased calcium (Ca^2+^) influx, Ca^2+^ overload, and subsequent activation of apoptotic signaling, elevated reactive oxygen species (ROS), mitochondrial dysfunction (ΔΨm), and altered NMDAR expression (particularly increased GluN2B), ultimately contributing to neuronal death and neurodegeneration. As a protective mechanism, phytochemicals can modulate NMDAR activity, reducing Ca^2+^ influx (partly via Mg^2+^-associated mechanisms), reducing the induction of apoptosis, decreasing ROS levels, restoring ΔΨm, and supporting neuroprotective NMDAR expression (reduced GluN2B and increased NR2A), thereby promoting neuronal survival and protection against NDDs.

## Figures and Tables

**Table 1:** Plant extracts and isolated phytochemicals that exhibited NMDA receptor-modulating effects in included studies.

References	Study Model	Plant Scientific Name/ Type of Extract	Concentration of Plant	Outcomes	Conclusions
Weichel et al., 1999 [15]	Hippocampal slices from Wistar rats and Wistar rats (in vivo)	Bilobalide, a constituent of *Ginkgo biloba*	1–100 µM in vitro and at 20 mg/kg i.p. in vivo	Decreased NMDA-induced choline release (IC_50_ = 2.3 µM). Decreased NMDA-induced PLA_2_ activation by NMDAR and Ca^2+^ influx via the prevention of the formation of *lyso*-PC (*p* < 0.05 vs. NMDA) and GPCh (*p* < 0.01 vs. NMDA). Antagonized the NMDA-induced increase of choline release in vivo. Suppressed NMDA-induced convulsions in vivo.	Bilobalide acts downstream of NMDAR activation but upstream of PLA_2_ activation to block choline release.
Zhang and Hu, 2001 [16]	Primary hippocampal neurons from SD rats	Huperzine A, an alkaloid isolated from the Chinese herb *Huperzia serrata*	100 μM	Reversibly inhibited NMDA-induced current (IC_50_ = 126 μM), was non-competitive, and showed neither ‘voltage-dependency’ nor ‘use-dependency’. IC_50_ values of huperzine A were not significantly altered by changing [glycine] (2–0.2 μM) pH (7.4–6.7), addition of Zn^2+^ (5 μM), or DTT (5 mM) to the external solution. The IC_50_ of huperzine A was increased from 146 μM to 321 μM with spermine (200 μM), indicative of interaction with one of the polyamine binding sites.	Huperzine A functions as a non-competitive antagonist of NMDAR by competitively interacting with one of the polyamine binding sites.
Kang et al., 2002 [17]	*Xenopus laevis* oocytes injected with total RNA from cortices or cerebelli from Wistar rats	Rhynchophylline and isorhynchophylline (tetracyclic oxindole alkaloids from *Uncaira* plant species)	1–100 μM	Inhibited NMDA-evoked currents in a concentration-dependent manner (*p* < 0.01 vs. NMDA + 3 μM glycine) (IC_50_ = 43.2 [26.0–72.0] and 48.3 [35.4–65.8] μM (mean [95% CI]) for rhynchophylline and isorhynchophylline, respectively. Reduced the NMDA-induced currents in a voltage-independent manner. Long-term exposure did not affect the suppression of NMDA currents, suggesting a non-use-dependent effect. Reduced the maximal current responses of NMDAR channels in Xenopus oocytes (*p* < 0.01). Reduced Emax values of the glycine response (*p* < 0.01). No interaction with the polyamine binding site, the Zn^2+^ site, the proton site, or the redox modulatory site on NMDARs.	Rhynchophylline and isorhynchophylline produce a concentration-dependent and reversible inhibition of NMDAR function in oocytes with similar potency, indicative of activity as noncompetitive antagonists, and this may contribute to the neuroprotective and anticonvulsant activity of the *Uncaira* species plant extracts.
Chatterjee et al., 2003 [18]	Primary hippocampal neurons from Wistar rats	*Ginkgo biloba* leaf extract, ginkgolides A, B, C, and J in EGb 761, bilobalide, and Ginkgolide M	Ginkgolides A, B, C J, and M and bilobalide at 10 µM	Bilobalide weakly inhibited the NMDAR-mediated current. Ginkgolide M blocked NMDAR-gated responses.	NMDA-gated depolarization could partly explain the seizure-preventing effects of bilobalide; it is also possible that the blocking actions of bilobalide on NMDAR partly contribute to this effect.
Sun et al., 2003 [19]	Primary cortical neurons from mice	Aqueous extract of the Traditional Chinese medicine (TCM): *Ligusticum chuanxiong* Hort. *Rehmannia glutinosa* Libosch. *Notopterygium incisium* Ting ex H. T. Chang. *Saiga tatarica* Linaeus. *Paeonia lactiflora* Pall. *Pheretima aspergillum* E. Perrier. *Scolopendra subspinipes* mutilans L. Koch. *Poria cocos* (Schw.) Wolf. *Saposhnikovia divaricata* (Turcz.) Schischk. *Bombyx mori* L. *Gentiana macrophylla* Pall. *Angelica pubescens* Maxim. f. biserrata Shan et Yuan. *Asarum heterotropoides* Fr. Var. mandshuricum (Maxim.) Kitag. *Crytotympana pustulata* Fabricius. *Gastrodia elata* Bl. *Achyrantes bidentata* Bl. *Carthamus tinctorius* L. *Buthus martensii* kirsch. *Salvia miltiorrhiza* Bge. *Scutellaria baicalensis* Georgi. *Uncaria rhynchophylla* (Miq.) Jacks. *Stephania tetrandria* S. Moore	1% (*v*/*v*)	Aqueous extract of *S. baicalensis*, *S. tetrandra*, *U. rhynchophylla,* and *S. miltiorrhiza* decreased NMDA-induced currents at a holding potential of −80 mV (*p* < 0.001 vs. NMDA), which was voltage-dependent, and with a magnitude of decrease lower than that predicted from their content of Mg^2+^. At a holding potential of +60 mV (depolarized potentials), the NMDA-evoked current was decreased by *U. rhynchophylla*, and this block was higher than predicted from its content of Mg^2+^ at negative holding potentials. *U. rhynchophylla* reduced the NMDA-induced decrease in cell viability in a concentration-dependent manner (*p* < 0.001 vs. NMDA).	Aqueous extracts of *S. baicalensis*, *S. tetrandra*, *S. miltiorrhiza*, and *U. rhynchophylla* partially blocked NMDA-evoked currents, and for *S. baicalensis*, *S. tetrandra*, and *S. miltiorrhiza* this may reflect the presence of Mg^2+^ in the extracts of these TCMs. In contrast, the extract of *U. rhynchophylla* blocked NMDA-evoked currents even at depolarized potentials and reduced the NMDA-induced decrease in neuronal cell viability.
Sun et al., 2005 [20]	Primary cortical mouse neurons	Aqueous extracts of dried roots of *Salvia miltiorrhiza* Bunge (Labiatae) (Traditional Chinese Medicine Danshen)	1 mg/mL	Blocked NMDA-evoked currents. Aqueous Danshen extracts contained ~9 mM magnesium. Free Mg^2+^ ions account for the NMDA antagonist activity.	Blockage of NMDA-evoked currents was voltage-dependent and showed a negative slope conductance reminiscent of the effect of Mg^2+^ ions.
Ban et al., (2006) [21]	Primary cortical neurons from SD rats	Methanolic extract of *Smilacis chinae* rhizome (SCR)	10, 30, and 50 µg/mL	Decreased Aβ (25–35)-induced loss of cell viability (*p* < 0.01 vs. Aβ). Decreased Aβ (25–35)-induced apoptosis (*p* < 0.01 vs. Aβ). Inhibited the Aβ (25–35)-induced cytosolic [Ca^2+^] increase. Decreased the Aβ (25–35)-induced elevation of L-Glu (*p* < 0.05 and *p* < 0.01 vs. Aβ). Reduced the Aβ (25–35)-induced increase in ROS (*p* < 0.01 vs. Aβ). Reduced the Aβ (25–35)-induced increase of caspase-3 (*p* < 0.05 vs. Aβ).	The neuroprotective effects of SCR against the pathological peptide Aβ (25–35) is mediated by reduced L-Glu production and lowering of the induction of excitotoxicity mechanisms.
Kumar et al., 2006 [22]	Primary cortical neurons and hippocampal slices from Wistar rats	Hyperforin, a constituent of St. John’s wort (*Hypericum perforatum*)	10 µM	Decreased the NMDA-induced Ca^2+^ influx (*p* < 0.01 vs. NMDA). Decreased the NMDA-induced release of choline.	Hyperforin could be neuroprotective via NMDAR antagonistic properties.
Ban et al., 2008 [23]	Primary rat cortical neurons and SD rats (in vivo)	Methanolic extract of *Smilacis chinae* rhizome (SCR) and oxyresveratrol and resveratrol isolated from the SCR	SCR (10, 30, and 50 µg/mL), oxyresveratrol (1, 10 µM), and resveratrol (10, 30, 50 µM)	SCR decreased NMDA-induced cell death (MTT assay, *p* < 0.01 vs. NMDA). SCR decreased NMDA-induced apoptosis (*p* < 0.01 vs. NMDA). Oxyresveratrol (*p* < 0.05, *p* < 0.01 vs. NMDA) and resveratrol (*p* < 0.01 vs. NMDA) decreased NMDA-induced cell death. SCR, oxyresveratrol, and resveratrol completely inhibited the NMDA-induced increase in [Ca^2+^]i. SCR, oxyresveratrol, and resveratrol reduced the NMDA-induced increase in ROS (*p* < 0.01 vs. NMDA). SCR reduced infarction in coronal sections in MCAO rats (*p* < 0.01 vs. vehicle-treated MCAO group). SCR reduced MCAO-induced neuronal cell damage.	The neuroprotective effects of SCR and its constituents oxyresveratrol and resveratrol reflect anti-excitotoxic (NMDAR) effects, so SCR may have a therapeutic role in NDDs such as stroke.
Nguyen et al., 2008 [24]	Primary cortical neurons from SD rats and SD rats (in vivo)	Methanolic extract from *Sanguisorbae* radix (SR), the root of *Sanguisorba officinalis* L. (Rosaceae) Catechin isolated from SR	SR at 10, 30, 50 µg/mL (in vitro) and 10 and 30 mg/kg (in vivo) Catechin at 10, 50, 100 µM (in vitro)	SR and catechin reduced the H_2_O_2_-induced decrease of cell viability in a concentration-dependent manner (*p* < 0.01 vs. H_2_O_2_). SR and catechin reduced the H_2_O_2_-induced increase in apoptosis in a concentration-dependent manner (*p* < 0.01 vs. H_2_O_2_). SR reduced the H_2_O_2_-induced increase of [Ca^2+^]i in a concentration-dependent manner. SR reduced the H_2_O_2_-induced elevation of L-Glu in a concentration-dependent manner (*p* < 0.01 vs. H_2_O_2_). SR reduced the H_2_O_2_-induced increase of ROS in a concentration-dependent manner (*p* < 0.01 vs. H_2_O_2_). SR reduced infarction and edema in MCAO rats (*p* < 0.05, *p* < 0.01 vs. vehicle-treated control group). SR reduced neurological deficit scores (*p* < 0.01 vs. vehicle-treated control group).	SR is neuroprotective by inhibiting the accumulation of extracellular L-Glu and NMDAR stimulation and associated increases in [Ca^2+^]i and production of ROS; therefore, the neuroprotective effect of SR against focal cerebral ischemic injury, such as that observed in stroke, reflects its anti-oxidative effects.
Pedersen et al., 2008 [25]	Mouse cortical wedge preparation from NMRI mice	Ethanolic extract from the leaves of *Searsia dentata* and *Searsia pyroides*	0.01–2.0 mg/mL	Inhibited SEDs in a concentration-dependent manner (ED_50_ = 0.62 [0.33; 1.16 (95% CI)] and 1.65 [1.17; 2.32 (95% CI)] (mg dry extract/mL) for *Searsia dentata* and *Searsia pyroides*, respectively. Inhibited the binding of NMDA antagonist [^3^H]-CGP39653 (IC_50_ = 0.48 [0.35; 0.66 (95% CI)] and 0.73 [0.53; 0.97 (95% CI)], (mg dry extract/mL) for *Searsia dentata* and *Searsia pyroides*, respectively. Decreased the amplitude of NMDA-induced depolarization (*p* < 0.05).	*Searsia pyroides* and *Searsia dentata* crude ethanolic extracts display NMDAR antagonistic effects.
Gu et al., 2009 [26]	Primary mouse cortical neurons and human embryonic kidney (HEK) 293 cells transfected with GluN1/GluN2A or GluN1/GluN2B subunits	Notoginsenoside R1 (NTR1), the main active ingredient in *Panax notoginseng*, a herbal medicine	0.1–10 μM	Decreased the L-Glu-induced loss of cell viability in a concentration-dependent manner (MTT assay: *p* < 0.05, and *p* < 0.01; LDH assay: *p* < 0.05, *p* < 0.01, and *p* < 0.001 vs. L-Glu alone). Decreased L-Glu-induced apoptosis in a concentration-dependent manner (*p* < 0.01 and *p* < 0.001 vs. L-Glu). Decreased L-Glu-induced [Ca^2+^]i in a concentration-dependent manner (*p* < 0.05, *p* < 0.001 vs. L-Glu alone). Decreased L-Glu-induced ROS in a concentration-dependent manner (*p* < 0.01 and *p* < 0.001 vs. L-Glu alone). Recovered decreased levels of Bcl-2 and increased levels of Bax in a concentration-dependent manner (*p* < 0.05, *p* < 0.01, and *p* < 0.001 vs. L-Glu alone). Decreased the L-Glu-induced increase in the MMP in a concentration-dependent manner (*p* < 0.05, *p* < 0.01 vs. L-Glu alone). Protected GluN1/GluN2B subunit expressing cells from cell death by 100 μM NMDA but not cells expressing GluN1/GluN2A subunits.	NTR1 may preferentially protect neurons from L-Glu excitotoxicity mediated by NMDAR composed of GluN1/GluN2B subunit assembly.
Zhang et al., 2010 [27]	Primary neurons from the cortex of SD rats	Hyperoside (flavonoid) compound isolated from *Rhododendron ponticum* L. leaves	0.1–10 μM	Decreased NMDA-induced loss of cell viability in a concentration-dependent manner (*p* < 0.05 and *p* < 0.01 vs. NMDA). Decreased NMDA-induced apoptosis (*p* < 0.01 vs. NMDA). Increased Bcl-2 expression (*p* < 0.05 vs. NMDA) and reduced Bax expression (*p* < 0.05 and *p* < 0.01 vs. NMDA). Reduced the NMDA-induced GluN2B subtype overexpression (*p* < 0.05 vs. NMDA).	Hyperoside is neuroprotective by inhibiting NMDA-evoked apoptosis through upregulation of Bcl-2 and reduced Bax expression and by reducing GluN2B expression.
Marchetti et al., 2011 [28]	Rat cerebellar granule cells and human embryonic kidney (HEK) 293 cells transfected with GluN1/GluN2A or GluN1/GluN2B subunits	Ethanolic extracts from the leaves of *Searsia pyroides*, *Searsia dentata*, and *Searsia glauca*	0.01, 0.1, and 1 mg/mL	Extracts decreased the current elicited by 500 μM NMDA in the presence of 30 μM glycine. For *Searsia pyroides* and *Searsia dentata*, this was up to 70% at 0.1 mg/mL with ED_50_s = 0.031 and 0.022 mg/mL, respectively. The effect of *Searsia glauca* was significantly less pronounced than the other extracts, both at 0.1 and at 1 mg/mL (*p* < 0.05). Extracts decreased NMDA-induced currents in HEK293 cells transfected with GluN1/GluN2A receptor channels by >80% for *Searsia pyroides* and *Searsia dentata*. The effect of *Searsia pyroides* was not mediated by the redox regulatory site. *Searsia pyroides* at 0.02 and 0.1 mg/mL decreased NMDA-induced [Ca^2+^] influx.	Ethanolic extracts from the leaves of *Searsia pyroides*, *Searsia dentata*, and *Searsia glauca* contain agents able to suppress NMDA-induced currents, and the extract from *Searsia pyroides* can also reduce the NMDA-induced increase in [Ca^2+^]i.
Kim et al., 2012 [29]	Primary cortical neurons from SD rats	Methanolic extract from the leaf and stem of *Vitis amurensis* (Vitaceae) and isolated (+)-ampelopsin A, γ-2-viniferin, and trans-ε-viniferin	*V. amurensis* methanolic extract (1, 10, and 50 μg/mL) (+)-ampelopsin A, γ-2-viniferin, and trans-ε-viniferin (0.5, 1, and 5 μM)	Extract and chemicals decreased L-Glu-induced loss of cell viability (*p* < 0.01 vs. L-Glu). Extract and chemicals reduced the L-Glu-induced decrease of BcL-2 and increase of Bax protein expression. Extract and chemicals decreased L-Glu-induced [Ca^2+]^i elevation. Extract and chemicals decreased the L-Glu-induced ROS in a concentration-dependent manner (*p* < 0.01 vs. L-Glu).	The *Vitis amurensis* extract and (+)-ampelopsin A, γ-2-viniferin, and trans-ε-viniferin inhibited L-Glu-induced neuronal death and induction of apoptosis, elevation of [Ca^2+^]i, and generation of ROS, suggesting that the neuroprotective effect of *V. amurensis* is (at least) partially attributed to these compounds.
Li et al., 2012 [30]	Primary cortical mouse neurons	Flax Lignan (purity > 98%), purchased from Shanghai PureOne Biotechnology	1 and 10 μM	Decreased NMDA-induced loss of cell viability at 1 μM for 24 h (77.4 ± 6.1%, *p* < 0.05 vs. NMDA alone) and 10 μM (85.2 ± 4.3%, *p* < 0.01 vs. NMDA alone). Decreased NMDA-induced apoptosis (1 and 10 μM) by 23.4 ± 4.2% and 9.8 ± 6.1%, respectively (*p* < 0.01 vs. NMDA alone). Decreased Bax expression (*p* < 0.05 vs. NMDA alone, 10 μM), increased Bcl-2 expression (*p* < 0.05, *p* < 0.01 versus NMDA alone, 10 μM), and increased the ratio of Bax/Bcl-2 (*p* < 0.05, *p* < 0.01 versus NMDA alone, 10 μM). Decreased NMDA induction of GluN2B subtype expression (*p* < 0.05 (1 μM) and *p* < 0.01 10 μM) vs. NMDA alone). Decreased NMDA-induced cytoplasmic Ca^2+^ levels.	Flax Lignan protected cortical neurons, reduced induction of apoptosis, inhibited NMDA-upregulated GluN2B expression, and did not interact directly with NMDA receptors to inhibit Ca^2+^ influx.
Yang et al., 2014 [31]	Primary cortical neuronal cultures from SD rats and synaptic forebrains from SD rats	Ethanolic extract from roots of *Scutellaria baicalensis*	1, 10, 25, 50, and 100 μg/mL	Decreased L-Glu and NMDA-induced loss of cell viability—LDH assays with IC_50_s of 60.01 and 28.60 μg/mL, respectively. Extract competitively inhibited the binding of [^3^H]MDL 105,519 (selective NMDAR glycine site antagonist) in a concentration-dependent manner (IC_50_ of 35.1 μg/mL). Extract inhibited the binding of [^3^H]MK-801 (NMDAR antagonist) to NMDAR in a concentration-dependent manner (IC_50_ of 65.1 μg/mL, *p* < 0.05 and *p* < 0.01 vs. control).	The extract exhibited neuroprotection against excitotoxic cell death mediated through the inhibition of NMDAR function through interaction with the glycine binding site.
Tian et al., 2015 [32]	Human neuroblastoma SH-SY5Y cells	1,5-*O*-dicaffeoyl-3-*O*-[4-malic acid methyl ester]-quinic acid (MQA), a natural derivative of caffeoylquinic acid, isolated from *Arctium lappa* L. roots	5–40 µM	Reduced the NMDA-induced decrease in cell viability (MTT assay, *p* < 0.05 and *p* < 0.01 vs. NMDA; LDH assay, *p* < 0.05 and *p* < 0.01 vs. NMDA). Decreased NMDA-induced apoptosis (*p* < 0.01 vs. NMDA). Reduced the NMDA-induced increase in [Ca^2+^]i. Reduced the NMDA-induced increase of the Bax/Bcl-2 ratio (*p* < 0.01 vs. NMDA), cytochrome c (*p* < 0.05 and *p* < 0.01 vs. NMDA), caspase-3 and caspase-9 protein and activities (*p* < 0.01 vs. NMDA). Decreased the levels of p-ERK1/2, p-p38, and p-JNK1/2 (*p* < 0.05 and *p* < 0.01 vs. NMDA). Restored phosphorylation of CREB, Akt, and GSK-3β reduced by NMDA treatment (*p* < 0.05 and *p* < 0.01 vs. NMDA). Reduced the NMDA-induced decrease in GluN2A expression and the NMDA-induced increase in GluN2B expression (*p* < 0.05 and *p* < 0.01 vs. NMDA).	The neuroprotective effects of MQA against NMDA-induced cell injury are mediated by inhibition of the activation of NMDARs.
Wang et al., 2016 [33]	C57BL/6 mice coronal brain slices and mice hippocampal neurons	Hydroxysafflor yellow A (HSYA), a compound extracted from *Carthamus tinctorius*	1–100 μM	Reduced the amplitude of NMDAR-mediated EPSCs (*p* < 0.01). Reversibly inhibited NMDAR EPSCs in a concentration-dependent manner (IC_50_ = 17.60 μM). Reduced the amplitude of OGD-induced NMDAR-mediated currents (*p* < 0.001). Suppressed the NMDAR-dependent OGD-induced iLTP in brain slices (*p* = 0.0003). Rescued the NMDA-induced [Ca^2+^]_i_ increase in a concentration-dependent manner (*p* < 0.001). Decreased NMDA-induced apoptosis in a concentration-dependent manner (*p* < 0.001 vs. NMDA). Decreased the NMDA-induced loss of cell viability in a concentration-dependent manner (LDH assay, *p* < 0.01 and *p* < 0.001 vs. NMDA). Reduced the NMDA-induced increase in the production of cleaved caspase-3 (*p* < 0.01 vs. NMDA). Reduced NMDA-induced mitochondria damage.	HSYA protects hippocampal neurons from excitotoxic damage through the inhibition of NMDARs.
Zhang et al., 2016 [34]	Primary hippocampal neurons from SD rats	YY-23, a compound derived from *Rhizoma anemarrhenae*	3 μM	Inhibited NMDA-induced current as a non-competitive antagonist (*p* < 0.01 vs. NMDA) (IC_50_ = 2.8 μM, 95% CI: 1.8 to 4.2 μM). Inhibited NMDA-induced currents in a voltage-independent or use-independent manner.	YY-23 acts as a non-competitive antagonist (allosteric modulator) of the NMDAR with fast blocking and unblocking characteristics and displayed neither ‘voltage–dependency’ nor ‘use-dependency’.
Lin et al., 2017 [35]	Primary cortical neurons from mice	Baicalein, present in *Scutellaria baricalensis*	4–14 μM	Baicalein reduced NMDA-induced depolarization via the NMDAR (*p* < 0.05 vs. NMDA).	Baicalein inhibits A-induced depolarization and possibly functions as an antagonist of AMPA and NMDARs.
Liu et al., 2019 [36]	Human SH-SY5Y neuroblastoma cells	Ethanolic extract of Anhua dark tea (produced from a crude green tea from leaves of *Camellia sinensis* var. *assamica*) 1–15 compounds isolated	5- 20 μM (compound 1 is 2*S*,3*R*-6-methoxycarbonylgallocatechin)	Decreased NMDA-induced loss of cell viability (*p* < 0.05 and *p* < 0.01 vs. NMDA). Decreased the NMDA-induced expression of GluN2B (*p* < 0.01). Increased p-Akt and p-ERK1/2 (*p* < 0.01 vs. NMDA). Decreased NMDA-induced apoptosis. Decreased NMDA-induced ROS (*p* < 0.05 vs. NMDA). Decreased the ratio of Bax/Bcl2 (*p* < 0.01 vs. NMDA). Decreased the expression of cleaved PARP (*p* < 0.05) and cleaved caspase-3 (*p* < 0.01) vs. NMDA.	Compounds present with neuroprotective effects via NMDAR inhibition, modulation of GluN2B expression, activation of PI3K/Akt signaling, and reduced NMDA induction of ROS and apoptosis.
Kuo et al., 2019 [37]	Primary mouse cortical neurons	Ginkgolide A extracted from *Ginkgo biloba*	100 and 300 µM	Decreased NMDA-induced depolarization (*p* < 0.05 vs. NMDA) at 300 µM but not at 100 µM.	Ginkgolide A has potential clinical application for the treatment of AD by reducing NMDAR activation.
Yang et al., 2020 [38]	Human neuroblastoma SH-SY5Y cells	4,5-*O*-dicaffeoyl-1-*O*-[4-malic acid methyl ester]-quinic acid (DCMQA), a natural caffeoylquinic acid derivative, isolated from *Arctium lappa* L. roots	10, 20, and 40 μM	Reduced the NMDA-induced decrease in cell viability in a concentration-dependent manner (MTT assay, *p* < 0.05 and *p* <0.01 vs. NMDA; LDH assay (*p* < 0.01 vs. NMDA). Decreased NMDA-induced apoptosis (*p* < 0.01 vs. NMDA). Decreased NMDA-induced cytosolic [Ca^2+^] influx. Decreased NMDA-induced ROS levels (*p* < 0.01 vs. NMDA). Reduced the NMDA-induced reduction in the MMP (*p* < 0.01 vs. NMDA). Reduced the NMDA-induced increase of the Bax/Bcl-2 ratio, (*p* < 0.01 vs. NMDA). Reduced the NMDA-induced release of cytochrome c (*p* < 0.01 vs. NMDA). Reduced the NMDA-induced increase in caspase-3 and caspase-9 expression (*p* < 0.01 vs. NMDA). Reduced the NMDA-induced decrease in GluN2A expression (*p* < 0.05 vs. NMDA) and the NMDA-induced increase in GluN2B expression (*p* < 0.01 vs. NMDA). Reduced the NMDA-induced expression of nNOS and PSD95 (*p* < 0.05 vs. NMDA). Increased the NMDA-induced reduction in CaMKII-α expression (*p* < 0.01 vs. NMDA).	DCMQA is neuroprotective against NMDA-induced neuronal damage by modulating NMDAR activity and downstream signaling.
Ding et al., 2021 [39]	Primary hippocampal neurons from SD rats	Bidentatide from aqueous extract of *Achyranthes bidentata* Blume	25–200 nM	Decreased NMDA-induced loss of cell viability in a concentration-dependent manner (*p* < 0.01–*p* < 0.001 vs. NMDA alone (50–200 nM). Decreased NMDA-induced [Ca^2+^]i in a concentration-dependent manner (*p* < 0.01 and *p* < 0.0001 vs. NMDA alone). 100 nM bidentatide and 100 nM NVP-AAM077 (GluN2A blocker) yielded a greater decrease in NMDA-evoked current compared to pretreatment with 100 nM NVPAAM077 alone (*p* < 0.01). 100 nM bidentatide ameliorated NMDA-induced MMP decrease (*p* < 0.01 vs. NMDA alone). Reduced NMDA-induced apoptosis (*p* < 0.01 vs. NMDA alone). Increased the Bcl-2/Bax ratio (*p* < 0.01 vs. NMDA alone) and reduced caspase 3 activity (*p* < 0.01 vs. NMDA alone).	Bidentatide can inhibit the activity of GluN2B-containing NMDA receptors.
ALNasser, et al., 2025 [40]	Differentiated human rhabdomyosarcoma TE671 cells	Acai berry *Euterpe oleracea* aqueous and ethanolic extracts	0.001–1000 µg/mL	Decreased the L-Glu-induced loss of cell viability in a concentration-dependent manner (MTT and LDH assays *p* < 0.05–*p* < 0.0001 vs. L-Glu). Decreased the L-Glu-induced reduction of cellular ATP levels (*p* < 0.05–*p* < 0.0001 vs. L-Glu alone). Improved the L-Glu-induced decline of the MMP (*p* < 0.05–*p* < 0.0001 vs. L-Glu alone). Decreased the L-Glu-induced increase in ROS levels (*p* < 0.05 and *p* < 0.0001 vs. L-Glu). Inhibited L-Glu and glycine-induced currents (*p* < 0.001 and *p* < 0.0001 vs. L-Glu and glycine).	Acai berry phytochemicals reduce L-Glu-induced NMDAR excitotoxicity and cytotoxicity.
Chang et al., 2025 [41]	SD rats (in vivo)	Epigoitrin (an alkaloid abundant in *Radix isatidis*)	10 and 50 mg/kg (15.4 and 77 µM, respectively)	10 mg/kg epigoitrin reduced KA-induced cortical cell degeneration (*p* < 0.0001 vs. KA alone). 50 mg/kg epigoitrin prevented KA-induced cortical cell degeneration. 50 mg/kg epigoitrin decreased p-DAPK1 levels and reduced the KA-induced expression of DAPK1 and phospho-GluN2B levels in rat cortex (*p* < 0.0001 vs. KA alone). 50 mg/kg epigoitrin decreased microglia and astrocyte activation in the cortex, decreased pro-inflammatory cytokine production, and increased anti-inflammatory cytokine production (*p* < 0.0001 vs. KA alone). 50 mg/kg epigoitrin decreased KA-induced ROS levels (*p* < 0.0001 vs. KA alone).	Epigoitrin protects cortical cells from KA-induced L-Glu-mediated excitotoxicity and neuroinflammation.

Abbreviations: AD: Alzheimer’s disease; Akt: alpha serine/threonine-protein kinase; Aβ25–35: amyloid beta (25–35); Ba^2+^: barium ions; Bax: Bcl-2 associated X protein; Bcl-2: B-cell lymphoma/leukemia-2; Ca^2+^: calcium ions; [Ca^2+^]i: intracellular Ca^2+^; CaMK II-α: Ca/calmodulin-dependent protein kinase II-α; CI: confidence interval; CREB: cAMP-response element binding protein; DNA: deoxyribonucleic acid; DTT: dithiothreitol; ED_50_: the 50% effective dose; Emax: maximum efficacy; EPSCs: excitatory postsynaptic currents; EtOH: ethanolic extract; GluN2A: N-methyl D-aspartate receptor subtype 2A; GluN2B: N-methyl D-aspartate receptor subtype 2B; GPCh: glycerophospho-choline; GSK-3β: Glycogen synthase kinase-3beta; H_2_O_2_: hydrogen peroxide; IC_50_: half-maximal inhibitory concentration; iLTP: ischemic long-term potentiation; i.p.: intraperitoneal; KA: kainic acid; LDH: lactate dehydrogenase; L-Glu: L-Glutamate; lyso-PC: lyso-phosphatidylcholine; MCAO: middle cerebral artery occlusion; Mg^2+^: magnesium ions; MMP: mitochondrial membrane potential; MTT: 3-(4,5-Dimethylthiazol-2-yl)-2,5-diphenyltetrazolium bromide; NDDs: neurodegenerative diseases; NMDA: N-Methyl-D-aspartic acid; NMDAR: NMDA-type (glutamate) receptor; NMRI: Naval Medical Research Institute; nNOS: neuronal nitric oxide synthase; NVP-AAM077: NMDA receptor antagonist selective for NMDA receptors containing GluN1/GluN2A subunits; OGD: oxygen–glucose deprivation; p-Akt: Phospho-alpha serine/threonine-protein kinase; p-DAPK1: Phospho-death-associated protein kinase 1; p-ERK1/2: Phospho-extracellular signal-regulated protein kinases 1 and 2; p-JNK1/2: Phospho-c-Jun N-terminal protein kinase; p-p38 MAPK: Phospho-p38 mitogen activated protein kinase; PARP: Cleaved-Poly (ADP-ribose) polymerase (PARP); PI: Propidium iodide; PI3K/Akt signaling; Phosphoinositide-3-kinase–protein kinase B/alpha serine/threonine-protein kinase signaling; PLA_2_: phospholipase A_2_; PSD95: protein-postsynaptic density protein 95; ROS: reactive oxygen species; SD: Sprague–Dawley; SEDs: spontaneous epileptiform discharges; Zn^2+^: zinc ions.

**Table 2:** In vivo studies: completed risk of bias matrix per study.

Study	Q1	Q2	Q3	Q4	Q5	Q6	Q7	Q8	Q9	Q10	Q11	Overall RoB Tier
Weichel et al., 1999 [15]	−	−	+	+	+	−	+	++	++	+	+	Low–moderate
Ban et al., 2008 [23]	−	−	+	+	+	−	−	+	+	+	+	Moderate
Nguyen et al., 2008 [24]	−	−	+	+	+	−	−	+	+	+	+	Moderate
Chang et al., 2025 [41]	+	−	+	+	+	−	+	++	+	+	+	Low–moderate

**Table 3:** In vitro studies: completed risk of bias matrix per study.

Study	Q1	Q2	Q3	Q4	Q5	Q6	Q7	Q8	Q9	Q10	Q11	Overall RoB Tier
Weichel et al., 1999 [15]	NA	NA	+	+	++	−	+	++	+	+	+	Low
Zhang and Hu, 2001 [16]	NA	NA	+	+	++	−	+	++	++	+	+	Low–moderate
Kang et al., 2002 [17]	NA	NA	+	+	++	−	+	++	++	+	+	Low–moderate
Chatterjee et al., 2003 [18]	NA	NA	+	+	++	−	+	++	++	+	+	Low–moderate
Sun et al., 2003 [19]	−	NA	+	+	++	−	+	+	++	+	+	Moderate
Sun et al., 2005 [20]	NA	NA	+	+	++	−	+	+	++	+	+	Low–moderate
Ban et al., 2006 [21]	−	NA	+	+	+	−	+	+	+	+	+	Moderate
Kumar et al., 2006 [22]	−	NA	+	+	+	−	+	++	+	+	+	Low–moderate
Ban et al., 2008 [23]	−	NA	+	+	+	−	+	+	+	+	+	Moderate
Nguyen et al., 2008 [24]	−	NA	+	+	+	−	+	+	+	+	+	Moderate
Pedersen et al., 2008 [25]	NA	NA	+	+	++	−	+	+	+	+	−	Moderate
Gu et al., 2009 [26]	+	NA	+	+	+	−	+	+	++	+	+	Low–moderate
Zhang et al., 2010 [27]	−	NA	+	+	+	−	+	+	+	+	+	Moderate
Marchetti et al., 2011 [28]	NA	NA	+	+	++	−	+	+	++	+	−	Moderate
Kim et al., 2012 [29]	−	NA	+	+	+	−	+	+	+	+	+	Moderate
Li et al., 2012 [30]	−	NA	+	+	+	−	+	+	+	+	+	Low–moderate
Yang et al., 2014 [31]	−	NA	+	+	+	−	+	+	+	+	+	Moderate
Tian et al., 2015 [32]	−	NA	+	+	+	−	+	++	+	+	+	Low–moderate
Wang et al., 2016 [33]	−	NA	+	+	++	−	+	++	++	+	+	Low
Zhang et al., 2016 [34]	NA	NA	+	+	++	−	+	++	++	+	++	Low
Lin et al., 2017 [35]	−	NA	+	+	+	−	+	+	+	+	+	Moderate
Liu et al., 2019 [36]	−	NA	+	+	+	−	+	+	+	+	+	Moderate
Kuo et al., 2019 [37]	+	NA	+	+	+	−	+	++	+	+	+	Low–moderate
Yang et al., 2020 [38]	−	NA	+	+	+	−	+	++	+	+	+	Low–moderate
Ding et al., 2021 [39]	−	NA	+	+	+	−	+	++	+	+	+	Low–moderate
ALNasser et al., 2025 [40]	+	NA	+	+	++	−	+	+	++	+	++	Low
Chang et al., 2025 [41]	−	NA	+	+	++	−	+	++	+	+	+	Low–moderate

Rating Scale/Legend: Definitely Low (++): Directly reported and adequate—method explicitly described and judged to minimize bias; Probably Low (+): Inferred to be adequate, or stated without full methodological detail; Probably High (−): Inferred to be inadequate, or insufficient detail raising concern; Not Applicable (NA): Question does not apply to this study design.

## Data Availability

The original contributions presented in this study are included in the article/supplementary material. Further inquiries can be directed to the corresponding author.

## Supplementary Materials

The following supporting information can be downloaded at https://www.mdpi.com/article/10.3390/cimb48060611/s1.
