# Supplementary material for: Phytochemicals as NMDA Receptor Inhibitors and Their Potential for Treating Excitotoxicity-Related Neurotoxicity: A Systematic Review
Source: Curr Issues Mol Biol. 2026 Jun 11;48(6):611. doi: 10.3390/cimb48060611 (PMC13298063; doi:10.3390/cimb48060611)
Supplement: Supplementary file 1 [file cimb-48-00611-s001.zip › cimb-4319797-supplementary.pdf]

Supplementary Materials

Table S1. PRISMA 2020 Checklist and PRISMA 2020 Abstract Checklist.

Section and Topic	Item #	Checklist item	Location where item is reported
**TITLE**			
Title	1	Identify the report as a systematic review.	Title
**ABSTRACT**			
Abstract	2	See the PRISMA 2020 for Abstracts checklist.	Abstract
**INTRODUCTION**			
Rationale	3	Describe the rationale for the review in the context of existing knowledge.	1. Introduction
Objectives	4	Provide an explicit statement of the objective(s) or question(s) the review addresses.	1.Introduction
**METHODS**			
Eligibility criteria	5	Specify the inclusion and exclusion criteria for the review and how studies were grouped for the syntheses.	Sections 2.4.1. and 2.4.2.
Information sources	6	Specify all databases, registers, websites, organisations, reference lists and other sources searched or consulted to identify studies. Specify the date when each source was last searched or consulted.	Section 2.3
Search strategy	7	Present the full search strategies for all databases, registers and websites, including any filters and limits used.	Section 2.3
Selection process	8	Specify the methods used to decide whether a study met the inclusion criteria of the review, including how many reviewers screened each record and each report retrieved, whether they worked independently, and if applicable, details of automation tools used in the process.	Section 2.4
Data collection process	9	Specify the methods used to collect data from reports, including how many reviewers collected data from each report, whether they worked independently, any processes for obtaining or confirming data from study investigators, and if applicable, details of automation tools used in the process.	Section 2.5
Data items	10a	List and define all outcomes for which data were sought. Specify whether all results that were compatible with each outcome domain in each study were sought (e.g. for all measures, time points, analyses), and if not, the methods used to decide which results to collect.	Section 2.5
	10b	List and define all other variables for which data were sought (e.g. participant and intervention characteristics, funding sources). Describe any assumptions made about any missing or unclear information.	Section 2.5
Study risk of bias assessment	11	Specify the methods used to assess risk of bias in the included studies, including details of the tool(s) used, how many reviewers assessed each study and whether they worked independently, and if applicable, details of automation tools used in the process.	Section 2.6
Effect measures	12	Specify for each outcome the effect measure(s) (e.g. risk ratio, mean difference) used in the synthesis or presentation of results.	N/A
Synthesis methods	13a	Describe the processes used to decide which studies were eligible for each synthesis (e.g. tabulating the study intervention characteristics and comparing against the planned groups for each synthesis (item #5)).	N/A

Section and Topic	Item #	Checklist item	Location where item is reported
	13b	Describe any methods required to prepare the data for presentation or synthesis, such as handling of missing summary statistics, or data conversions.	N/A
	13c	Describe any methods used to tabulate or visually display results of individual studies and syntheses.	
	13d	Describe any methods used to synthesize results and provide a rationale for the choice(s). If meta-analysis was performed, describe the model(s), method(s) to identify the presence and extent of statistical heterogeneity, and software package(s) used.	
	13e	Describe any methods used to explore possible causes of heterogeneity among study results (e.g. subgroup analysis, meta-regression).	
	13f	Describe any sensitivity analyses conducted to assess robustness of the synthesized results.	
Reporting bias assessment	14	Describe any methods used to assess risk of bias due to missing results in a synthesis (arising from reporting biases).	
Certainty assessment	15	Describe any methods used to assess certainty (or confidence) in the body of evidence for an outcome.	
**RESULTS**			
Study selection	16a	Describe the results of the search and selection process, from the number of records identified in the search to the number of studies included in the review, ideally using a flow diagram.	3.Results
	16b	Cite studies that might appear to meet the inclusion criteria, but which were excluded, and explain why they were excluded.	3. Results
Study characteristics	17	Cite each included study and present its characteristics.	3.Results
Risk of bias in studies	18	Present assessments of risk of bias for each included study.	Section 3.9
Results of individual studies	19	For all outcomes, present, for each study: (a) summary statistics for each group (where appropriate) and (b) an effect estimate and its precision (e.g. confidence/credible interval), ideally using structured tables or plots.	N/A
Results of syntheses	20a	For each synthesis, briefly summarise the characteristics and risk of bias among contributing studies.	Section 3.9
	20b	Present results of all statistical syntheses conducted. If meta-analysis was done, present for each the summary estimate and its precision (e.g. confidence/credible interval) and measures of statistical heterogeneity. If comparing groups, describe the direction of the effect.	N/A
	20c	Present results of all investigations of possible causes of heterogeneity among study results.	3.Results
	20d	Present results of all sensitivity analyses conducted to assess the robustness of the synthesized results.	3.Results
Reporting biases	21	Present assessments of risk of bias due to missing results (arising from reporting biases) for each synthesis assessed.	Section 3.9
Certainty of evidence	22	Present assessments of certainty (or confidence) in the body of evidence for each outcome assessed.	Section 3.9
**DISCUSSION**			
Discussion	23a	Provide a general interpretation of the results in the context of other evidence.	4.Discussion
	23b	Discuss any limitations of the evidence included in the review.	Section 4.8
	23c	Discuss any limitations of the review processes used.	Section 4.8

Section and Topic	Item #	Checklist item	Location where item is reported
	23d	Discuss implications of the results for practice, policy, and future research.	Section 4.8
**OTHER INFORMATION**			
Registration and protocol	24a	Provide registration information for the review, including register name and registration number, or state that the review was not registered.	Abstract and Section 2.1
	24b	Indicate where the review protocol can be accessed, or state that a protocol was not prepared.	N/A
	24c	Describe and explain any amendments to information provided at registration or in the protocol.	N/A
Support	25	Describe sources of financial or non-financial support for the review, and the role of the funders or sponsors in the review.	Funding statement
Competing interests	26	Declare any competing interests of review authors.	Conflicts of interest statement
Availability of data, code and other materials	27	Report which of the following are publicly available and where they can be found: template data collection forms; data extracted from included studies; data used for all analyses; analytic code; any other materials used in the review.	If applicable, available from the first author

Section and Topic	Item #	Checklist item	Reported (Yes/No)
**TITLE**			
Title	1	Identify the report as a systematic review.	Yes
**BACKGROUND**			
Objectives	2	Provide an explicit statement of the main objective(s) or question(s) the review addresses.	Yes
**METHODS**			
Eligibility criteria	3	Specify the inclusion and exclusion criteria for the review.	Yes
Information sources	4	Specify the information sources (e.g. databases, registers) used to identify studies and the date when each was last searched.	Yes
Risk of bias	5	Specify the methods used to assess risk of bias in the included studies.	Yes
Synthesis of results	6	Specify the methods used to present and synthesise results.	Yes
**RESULTS**			
Included studies	7	Give the total number of included studies and participants and summarise relevant characteristics of studies.	Yes
Synthesis of results	8	Present results for main outcomes, preferably indicating the number of included studies and participants for	Yes

Section and Topic	Item #	Checklist item	Location where item is reported
		each. If meta-analysis was done, report the summary estimate and confidence/credible interval. If comparing groups, indicate the direction of the effect (i.e. which group is favoured).	
**DISCUSSION**			
Limitations of evidence	9	Provide a brief summary of the limitations of the evidence included in the review (e.g. study risk of bias, inconsistency and imprecision).	Yes
Interpretation	10	Provide a general interpretation of the results and important implications.	Yes
**OTHER**			
Funding	11	Specify the primary source of funding for the review.	N/A
Registration	12	Provide the register name and registration number.	Yes for registration number

From: Page MJ, McKenzie JE, Bossuyt PM, Boutron I, Hoffmann TC, Mulrow CD, et al. The PRISMA 2020 statement: an updated guideline for reporting systematic reviews. BMJ 2021;372:n71. doi: 10.1136/bmj.n71. This work is licensed under CC BY 4.0. To view a copy of this license, visit https://creativecommons.org/licenses/by/4.0/.

OHAT Risk of Bias Assessment Tool — Modified.

Neuroprotective / NMDA-receptor–modulating phytochemicals and plant extracts

11 questions across 7 bias domains — applied to in vivo (animal) and in vitro (cell-based) studies

This tool uses the standard OHAT/NTP risk of bias framework (Office of Health Assessment and Translation, U.S. National Toxicology Program): 11 signalling questions grouped into seven bias domains. The standard OHAT wording is retained, and a modified question column re-frames each item specifically for NMDA-receptor–modulating phytochemical and plant-extract studies. The same question set is applied to both in vivo and in vitro studies.

Table S2. Question Key (Q1–Q11).

#	Domain	Standard OHAT signalling question
**1**	Selection bias	Was administered dose or exposure level adequately randomized?
**2**	Selection bias	Was allocation to study groups adequately concealed?
**3**	Selection bias	Did selection of study participants result in appropriate comparison groups?
**4**	Confounding bias	Did the study design or analysis account for important confounding and modifying variables?
**5**	Performance bias	Were experimental conditions identical across study groups?

#	Domain	Standard OHAT signalling question
6	Performance bias	Were the research personnel and human subjects blinded to the study group during the study?
7	Attrition / exclusion bias	Were outcome data complete without attrition or exclusion from analysis?
8	Detection bias	Can we be confident in the exposure characterization?
9	Detection bias	Can we be confident in the outcome assessment?
10	Selective reporting bias	Were all measured outcomes reported?
11	Other sources of bias	Were there no other potential threats to internal validity (appropriate statistics; adherence to protocol)?

Table S3. Part A — In Vivo (Animal) Studies: Domain Definitions & Modified Questions.

Bias Domain	#	Standard OHAT question	Modified RoB question applied to NMDA-modulating studies	Justification / application
**Selection bias**	1	Was administered dose or exposure level adequately randomized?	Were animals randomly assigned to phytochemical dose, vehicle, and the excitotoxic/NMDA challenge (e.g., NMDA, glutamate, kainate, ischemia/MCAO)?	Animals should be randomly assigned to extract/phytochemical dose levels, vehicle, and the excitotoxic challenge. A described random component balances baseline neurological status across arms.
**Selection bias**	2	Was allocation to study groups adequately concealed?	Was the assignment of animals to neuroprotectant vs. insult groups concealed from the person allocating them?	The person assigning animals to neuroprotective vs. insult groups should be unable to foresee the assignment, preventing selective placement.
**Selection bias**	3	Did selection of study participants result in appropriate comparison groups?	Were comparison groups matched for species, strain, sex, age and weight and drawn from the same source?	Groups should be comparable for species, strain, sex, age and weight, drawn from the same source, so differences in outcome reflect the intervention not the animals selected.
**Confounding bias**	4	Did the study design or analysis account for important confounding and modifying variables?	Were extract/phytochemical characteristics (botanical source, plant part, solvent, standardized marker, dose) and the timing of the insult relative to dosing controlled or adjusted for?	Key confounders include extract characterization and the timing of the insult relative to dosing. These should be controlled or adjusted for.
**Performance bias**	5	Were experimental conditions identical across study groups?	Were husbandry and the excitotoxic challenge (NMDA/glutamate dose, route, timing) identical across groups so the phytochemical was the only systematic difference?	Husbandry and the excitotoxic challenge must be uniform so the phytochemical is the only systematic difference between groups.
**Performance bias**	6	Were the research personnel and human subjects blinded to the study group during the study?	Were caregivers and assessors blinded during dosing and behavioural/histological readouts (neurological scoring, water maze, rotarod, seizure scoring)?	Caregivers/investigators should be blinded during dosing and behavioural testing, since these endpoints are sensitive to handler expectation. (No human subjects — applies to personnel.)

Bias Domain	#	Standard OHAT question	Modified RoB question applied to NMDA-modulating studies	Justification / application
**Attrition / exclusion bias**	**7**	Were outcome data complete without attrition or exclusion from analysis?	Were all animals accounted for, with insult-related deaths and exclusions explained and balanced across groups?	All animals should be accounted for; deaths from the excitotoxic insult and any exclusions must be explained and balanced across groups, as unequal attrition biases neuroprotection estimates.
**Detection bias**	**8**	Can we be confident in the exposure characterization?	Was the phytochemical/extract authenticated and standardized (botanical source, solvent, % active constituent, verified dose/purity)?	The extract/phytochemical should be authenticated and standardized; poorly characterized exposure makes effects non-attributable.
**Detection bias**	**9**	Can we be confident in the outcome assessment?	Were neuroprotection outcomes (infarct volume, neuronal counts/CA1, NR1/NR2B expression, behaviour) measured with validated, blinded methods applied identically across groups?	Outcomes should be measured with validated methods by blinded assessors applied identically across groups.
**Selective reporting bias**	**10**	Were all measured outcomes reported?	Were all measured outcomes, all tested doses, and null/non-protective results reported?	All pre-specified outcomes, all tested doses, and null results should be reported, preventing selective emphasis on protective findings.
**Other sources of bias**	**11**	Were there no other potential threats to internal validity (appropriate statistics; adherence to protocol)?	Were statistics appropriate (dose-response, multiple-comparison correction), sample size justified, protocol followed, and conflicts/funding declared?	Statistics should be appropriate, sample size justified, protocol adhered to, and conflicts/funding declared.

Table S4. Part B — In Vitro Studies: Domain Definitions & Modified Questions.

Bias Domain	#	Standard OHAT question	Modified RoB question applied to NMDA-modulating studies	Justification / application
**Selection bias**	**1**	Was administered dose or exposure level adequately randomized?	Were cells/wells randomly allocated to phytochemical concentrations, vehicle (DMSO), the NMDA/glutamate insult, and reference antagonists (MK-801, memantine, AP5)?	Cells/wells should be randomly allocated to concentrations, vehicle, the excitotoxic challenge and reference groups, reducing systematic plate/well differences.
**Selection bias**	**2**	Was allocation to study groups adequately concealed?	Was treatment identity concealed where feasible (e.g., coded plates)? (Mark NA for automated dispensing or single-cell/oocyte recording.)	Treatment identity should be concealed where feasible; record Not Applicable with rationale where automated dispensing or single-cell recording makes this irrelevant.
**Selection bias**	**3**	Did selection of study participants result in appropriate comparison groups?	Were the cell model (primary cortical/hippocampal neurons, SH-SY5Y, HEK-GluN, oocytes) and passage number consistent across groups?	The cell model and passage number should be consistent across groups, since cell type strongly affects NMDA-receptor expression.

Bias Domain	#	Standard OHAT question	Modified RoB question applied to NMDA-modulating studies	Justification / application
**Confounding bias**	**4**	Did the study design or analysis account for important confounding and modifying variables?	Were extract/compound characteristics (source, solvent, standardized constituent, purity) and culture conditions/passage controlled?	Control for extract characterization, passage number and culture conditions; these confound the neuroprotective signal if uncontrolled.
**Performance bias**	**5**	Were experimental conditions identical across study groups?	Were culture conditions and the NMDA challenge (agonist concentration, exposure time, Mg^{2+}-free/glycine conditions, DMSO, reagent lots) identical across groups?	Culture conditions and the excitotoxic challenge must be identical, as NMDA activation is highly sensitive to these.
**Performance bias**	**6**	Were the research personnel and human subjects blinded to the study group during the study?	Was the operator blinded to treatment identity where feasible? (Mark NA where not practical.)	The operator should be blinded to treatment identity where feasible; mark Not Applicable with rationale where not practical.
**Attrition / exclusion bias**	**7**	Were outcome data complete without attrition or exclusion from analysis?	Were all replicates/wells accounted for, with excluded wells (contamination, edge effects, failed dye loading) explained?	All replicates/wells should be accounted for; unexplained exclusion biases viability/IC_{50}/EC_{50} estimates.
**Detection bias**	**8**	Can we be confident in the exposure characterization?	Was the phytochemical/extract authenticated and standardized (source, solvent, % active constituent, purity, verified concentration), and intrinsic cytotoxicity characterized?	The extract/phytochemical should be authenticated and standardized, and intrinsic cytotoxicity characterized.
**Detection bias**	**9**	Can we be confident in the outcome assessment?	Were outcomes (MTT, LDH, Ca^{2+} via Fura-2/Fluo-4, patch-clamp NMDA current, ROS) read by blinded assessors or objective instruments with validated assays and positive controls?	Outcomes should be read by blinded assessors or objective instruments, with calibrated equipment, validated assays, and positive controls (MK-801, memantine, AP5).
**Selective reporting bias**	**10**	Were all measured outcomes reported?	Were all tested concentrations and endpoints reported, including non-protective doses and intrinsic cytotoxicity?	All tested concentrations and endpoints — including non-protective doses and intrinsic cytotoxicity — should be reported.
**Other sources of bias**	**11**	Were there no other potential threats to internal validity (appropriate statistics; adherence to protocol)?	Were biological vs. technical replicates clearly defined and adequate (≥3 independent experiments), statistics appropriate, and conflicts declared?	Biological vs. technical replicates should be clearly defined and adequate, statistics appropriate, protocol followed, and conflicts declared.
